# *Lactobacillus johnsonii* mediates the protective effects of pristimerin against ulcerative colitis and concomitant liver injury through remodeling hepatic lipid metabolism via LXRα–SCD1 axis

**DOI:** 10.1080/19490976.2026.2701349

**Published:** 2026-07-17

**Authors:** Yan Cheng, Zhanxuan E. Wu, Ruoyue Huang, Qingmei Li, Dongmei Yan, Yuqi Chen, WeiFeng Zhu, Fei Li

**Affiliations:** a Department of Gastroenterology & Hepatology, Laboratory of Hepato-intestinal Diseases and Metabolism, Frontiers Science Center for Disease-related Molecular Network, West China Hospital, Sichuan University, Chengdu, China; b School of Pharmacy, Jiangxi University of Chinese Medicine, Nanchang, China; c Academician Workstation, Jiangxi University of Chinese Medicine, Nanchang, China; d Key Laboratory of Modern Preparation of Traditional Chinese Medicine, Jiangxi University of Chinese Medicine, Nanchang, China

**Keywords:** *Lactobacillus johnsonii*, pristimerin, ulcerative colitis, liver injury, gut–liver axis

## Abstract

Ulcerative colitis (UC) is a systemic disease that can involve multiple organs, and hepatobiliary diseases in UC patients are frequently observed. However, the pathogenesis of UC and its associated hepatobiliary complications remains elusive, and limited therapeutic options are available. This study revealed that disrupted hepatic lipid metabolism plays a pivotal role in driving the progression of UC and its extraintestinal hepatobiliary manifestations. Mechanistically, colitis-elevated circulating endogenous corticosterone (CORT) mediates the downregulation of hepatic LXRα–SCD1 signaling, resulting in diminished monounsaturated fatty acid (MUFA), reduced unsaturated lysophospholipids, and the accumulation of alkyl lysophospholipids, ceramide and hexosylceramide. These alterations contribute to liver lipotoxicity and, in turn, exacerbate colitis. A similar lipid profile is observed in UC patients. Importantly, pristimerin, a natural compound structurally similar to the star molecule celastrol, has been demonstrated to alleviate UC and concomitant liver injury by remodeling hepatic lipid metabolism in a microbiota-dependent manner. The gut commensal *Lactobacillus johnsonii* mediates the effects of PSM by activating hepatic LXRα–SCD1 signaling and increasing the potential anti-inflammation lipid species LPC20:2 and LPC20:3. This investigation suggests a novel therapeutic strategy for UC and associated liver injury based on the *L. johnsonii*-hepatic LXRα–SCD1 axis. This study also opens new avenues for mechanistic exploration of systemic diseases and therapeutic strategies of multi-organ comorbidity.

## Graphical abstract

**Figure:**
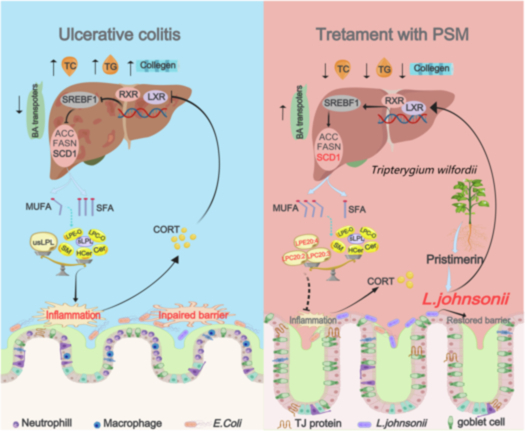

## Introduction

Ulcerative colitis (UC) is a chronic relapsing and remitting inflammatory bowel disease (IBD) characterized by abdominal pain, diarrhea, rectal bleeding and weight loss. As the incidence and prevalence of UC are increasing globally, its pathogenesis and therapy remain key areas of research interest.^1^ Currently, there is no curative therapy for UC. The treatment goal is to control inflammation and alleviate symptoms. Medications include the front-line therapy 5-amino-salicylates (5-ASA), while second-line therapies are corticosteroids, immunomodulators and biologics.^2^ ^,^ ^3^ However, owing to the limited efficacy and side effects of existing treatments, the exploration of novel, effective and safe treatment strategies continue to attract growing interest.^4^

UC is a systemic disease that can involve multiple organs. Extraintestinal manifestations (EIMs) are frequently observed, typically involving the joints, eyes, hepatobiliary tract and skin,^5^ which may eventually lead to severe complications and impair patients' prognosis.^6^ Hepatobiliary manifestations are disorders that affect the hepatobiliary system, including primary sclerosing cholangitis (PSC), non-alcoholic fatty liver disease (NAFLD), hepatic fibrosis, cholangiocarcinoma, and cholelithiasis, etc.^7^ In recent years, biologics such as anti-TNF (infliximab, IFX), anti-integrin α4β7 (vedolizumab, VDZ) and anti-interleukin12/23 (ustekinumab, UST) agents have been frequently employed for UC-associated NAFLD.^8^ ^,^ ^9^ Ursodeoxycholic acid (UDCA) has been used for concomitant UC and PSC.^10^ Owing to the lack of rigorous “evidence-based” data supporting the efficacy and various adverse effects, these therapeutic approaches should be carefully considered until further research specifically clarifies their roles in managing the hepatobiliary complications of UC patients.^9^ ^,^ ^11^

Given that numerous clinical needs in the management of UC and its hepatobiliary complications remain unmet, it is imperative to identify promising therapeutic targets and develop multi-organ strategies for these comorbidities. However, the etiology and pathogenesis of UC and associated hepatobiliary disease remain unclear. One extensively studied underlying mechanism is based on the gut‒liver axis. Specifically, liver injury in UC has been described as a direct consequence of a sustained, extended inflammatory response triggered by gut bacterial and viral endotoxins, such as bile acid (BA), lipopolysaccharide (LPS) and lactoferrin.^12-14^ Perturbation of gut‒liver crosstalk in various liver diseases has been demonstrated by clinical evidence.^15^ Likewise, as the interaction between the gut and liver is bidirectional, liver secretions also play crucial roles in intestinal homeostasis. Consequently, disruption of hepatic homeostasis and metabolism aggravates intestinal inflammation.^16-18^

Lipid metabolic disorders are very common in UC patients, particularly substantial shifts in phospholipid and sphingolipid metabolism.^19^ ^,^ ^20^ Overall, hepatic lipid metabolism, including lipogenesis, reverse cholesterol transport, and lipid oxidation mediated by LXRα-dependent pathways, is disrupted in DSS-induced colitis.^21^ Stearoyl-CoA desaturase-1 (SCD1) is a downstream target gene of LXRα and a key enzyme for mono-unsaturated fatty acid (MUFA) synthesis. SCD1 deficiency induced the production of ceramide, which is the central lipid building block of sphingolipids.^22^ Inhibition of SCD1 dramatically exacerbated colonic inflammation in colitis mice.^17^ We therefore speculated that hepatic de novo lipid synthesis mediated by LXRα–SCD1 signaling pathway may play a crucial role in the progression of UC and associated liver injury. In addition, UC patients with depression have been reported to exhibit abnormal cortisol levels, which can lead to metabolic dysregulation.^23^ In rodent models, increased corticosterone (CORT) drives the progression of NAFLD.^24^ Downregulation of LXRα caused by CORT induction has been observed in liver of juvenile chickens.^25^ Taken together, we hypothesized that CORT may exert an effect on hepatic LXRα–SCD1 signaling in mice with UC and concomitant liver steatosis.

**Figure 1.**
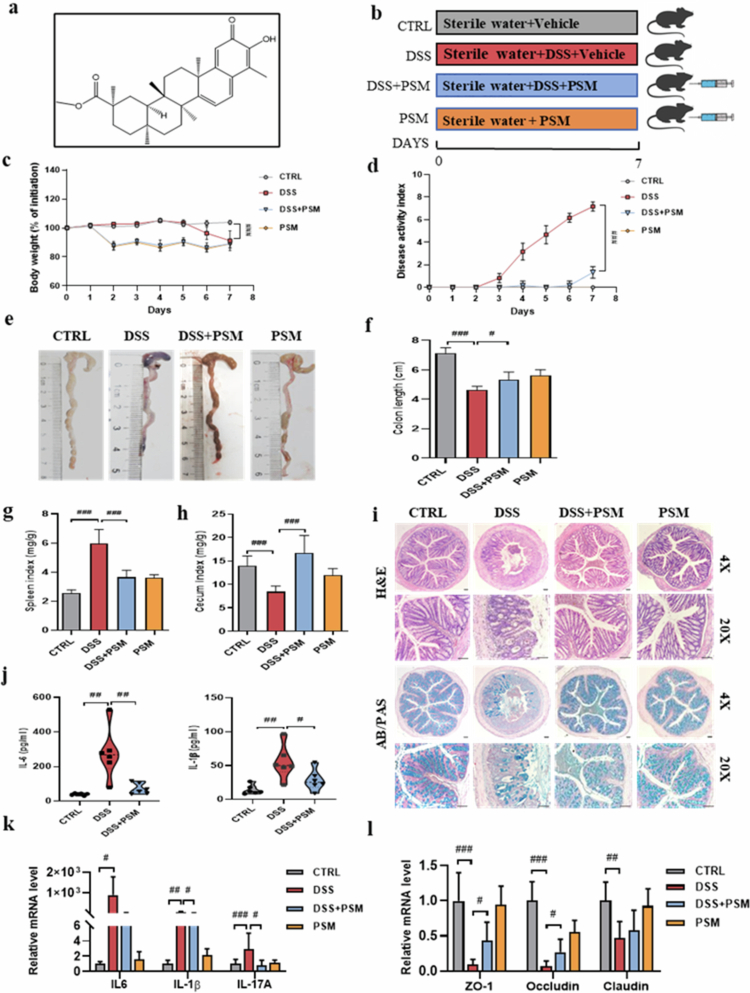
PSM alleviates DSS-induced colitis. (a) Molecular structure of pristimerin. (b) Schematic diagram of the mouse experiment. (c) Body weight changes throughout the experiment (*n*=6). (d) Disease activity index (DAI) during the experiment (*n*=6). (e) Representative images of the colon on day 8. (f) Colon length on day 8 (*n*=6). (g) Spleen index (ratio of spleen weight to body weight) (*n*=6). (h) Cecum index (ratio of cecum weight to body weight) (*n*=6). (i) Representative H&E and AB/PAS staining of distal colon sections (scale bar, 100 µm) (*n*=3). H&E staining showed increased inflammatory cell infiltration, AB/PAS staining showed a loss of goblet cells in distal colon tissue. (j) Levels of IL-6 and IL-1β in the serum (*n*=6). (k) Relative gene expression of IL-6, IL-1β and IL-17A in the colon (*n*=5-6). (l) Relative gene expression of ZO-1, occludin and claudin in the colon (*n*=6). #*p* < 0.05, ##*p* < 0.01, ###*p* < 0.001.

Natural compounds have been a major source for drug discovery.^1^ ^,^ ^26^ Interestingly, some natural compounds, such as cryptotanshinone and celastrol, can ameliorate colitis by modulating host lipid metabolism and the gut microbiota.^27^ ^,^ ^28^ Pristimerin (PSM, Figure 1a) is a pentacyclic triterpene isolated from the herbal plant *Tripterygium wilfordii* Hook. f. It is a natural compound structurally similar to the star molecule celastrol and also possesses various pharmacological activities. A recent study revealed that it exerts anti-colitis activity by modulating the gut microbiota balance and host lipid metabolism.^29^ However, it remains unclear what is the pivotal microbe it targets and the specific mechanisms by which it regulates lipid metabolism.

In this study, we demonstrated that PSM attenuates DSS-induced experimental UC and associated liver injury in a gut microbiota-dependent manner. The gut commensal *L. johnsonii* mediates the protective effects of PSM against this comorbidity by remodeling hepatic lipid metabolism via activation of the LXRα‒SCD1 signaling pathway. This finding indicates an underlying mechanism involving the microbiota‒liver‒gut axis in the pharmacological activity of PSM and paves the way for exploring *L. johnsonii* as a novel multi-organ therapeutic target for UC and concomitant hepatic diseases.

## Materials and methods

### Mouse experiments

All animal protocols were approved by the Animal Care and Use Committee of West China Hospital, Sichuan University (Approval ID: 20220226032). Male C57BL/6J mice (6–8 weeks old) were purchased from GemPharmatech Co., Ltd. (Jiangsu, China). 6–8 weeks-old male 129/Sv wild-type mice (WT) and 129/Sv *Pparα*-null mice were used to evaluate the function of PPARα. The method for creating *Pparα*-null mice have been described previously.^30^ Mice were groups-housed in individually ventilated cages under a standard 12 h light/dark cycle with a relative humidity of 40%–60% at a constant temperature of 22 °C. All the mice had ad libitum access to food and water during the experiments. Colitis was induced by the addition of drinking water containing 3.0% (w/v) DSS for 7 d.

*Animal experiment 1:* PSM alleviated UC and associated liver injury study. Male C57BL/6J mice were randomly divided into four groups (*n*=6): (1) CTRL, (2) DSS, (3) DSS+PSM (MCE, cat#1258-84-0), and (4) PSM. Regular sterilized water was given to CTRL and PSM groups, and 3% (w/v) DSS water was administered to DSS and DSS+PSM groups. Based on the previous literature and our preliminary experiment,^31^ ^,^ ^32^ a dosage of 0.5 mg/kg of PSM was selected. Over the duration of 7 d, mice in DSS + PSM and PSM groups were treated with PSM (0.5 mg/kg) by intraperitoneally injection (i.p. injection) once every 2 d, 4 doses in total. The CTRL and DSS groups were concurrently administered vehicle (1% DMSO + 2% Tween-80 sterilized water) by i.p. injection.

*Animal experiment 2:* ABX treatment depleted the gut microbiota study. Male C57BL/6J mice were randomly assigned to three groups (*n *= 6): (1) CTRL, (2) PSM, and (3) ABX+PSM. Over the three weeks duration, regular sterilized water was given to the CTRL and PSM groups, and the mice in the ABX+PSM group were treated with antibiotic cocktails (ampicillin, 0.25 mg/ml; metronidazole, 0.25 mg/ml; neomycin, 0.25 mg/ml; vancomycin, 0.125 mg/ml). From day 15 to day 21, the mice in PSM and ABX+PSM groups were treated with PSM (0.5 mg/kg) by i.p. injection every 2 d, 4 doses in total. The CTRL group was concurrently administered vehicle (1% DMSO + 2% Tween-80 sterilized water) via i.p. injection.

*Animal experiment 3: L. johnsonii* alleviated UC and associated liver injury study. Male C57BL/6J mice were randomly assigned into four groups (*n*=6): (1) CTRL, (2) DSS, (3) DSS+Ljsup, and (4) Ljsup. Over the three weeks duration, regular sterilized water was given to the CTRL and Ljsup groups. For the DSS and DSS+Ljsup groups, the mice were given regular sterilized water for the first two weeks, followed by 3% (w/v) DSS water for another week. The mice in DSS+Ljsup and Ljsup groups were treated with 0.2 mL culture supernatant of *L. johnsonii* (Ljsup) by daily oral gavage for three weeks, and the CTRL and DSS groups were concurrently given vehicle (Man–Rogosa–Sharpe (MRS) medium).

*Animal experiment 4:* SR9238 abolished anti-colitis and liver protection effects of *L. johnsonii* study. Male C57BL/6J mice were randomly assigned to three groups (*n*=6): (1) DSS, (2) Ljsup, and (3) SR9238 (MCE, cat#1416153-62-2). Over the three weeks duration, the mice in Ljsup and SR9238 group were treated with Ljsup by daily oral gavage for three weeks, and the DSS group was concurrently given vehicle (MRS medium). From day 8 to 21, the mice in the SR9238 group were treated with SR9238 (30 mg/kg) by daily i.p. injection, and the DSS and Ljsup groups were concurrently given vehicle (10% DMSO + 10%Tween-80 sterilized water). From day 15 to 21, 3% (w/v) DSS water was administered to mice in all the three groups.

*Animal experiment 5:* CORT inhibited hepatic LXRα–SCD1 signaling to aggravate colitis and associated liver injury study. Male C57BL/6J mice were randomly assigned to two groups (*n*=6): (1) DSS, (2) DSS+CORT (Caymen, cat#: 50-22-6). Over the duration of 12 d, the mice in the DSS+CORT group were daily subcutaneously injected with CORT (10 mg/kg), and the DSS group was concurrently given vehicle (15% ethanol). From day 5 to day 12, 3% (w/v) DSS water was administered to the mice in both groups. Male C57BL/6J mice were randomly assigned to two groups (*n*=6): (1) DSS+SR9243, (2) DSS+SR9243 + CORT (MCE, cat# HY-16972). Over the duration of 12 d, mice in DSS+SR9243+CORT group were injected with CORT (10 mg/kg) subcutaneously, and SR9243 (30 mg/kg) intraperitoneally every day. The DSS + SR9243 group was concurrently treated with SR9243 and vehicle (10% DMSO + 10% Tween-80 sterilized water). From day 5 to 12, 3% (w/v) DSS water was administered to the mice in both groups.

*Animal experiment 6*: PPARα KO-induced increase in hepatic SCD1 expression protected against colitis study, male 129/Sv wild-type mice (WT) were randomly classified into three groups (*n*=5): (1) WT-CTRL, (2) WT-DSS, and (3) WT-DSS+Wy14643 (MCE, cat#:50892-23-4). Male 129/Sv Pparα-null mice were randomly assigned to two groups (*n*=4): (4) KO-CTRL, (5) KO-DSS. Over one week duration, mice in the WT-CTRL and KO-CTRL groups were given regular sterilized water, and those in the other three groups were administered to 3% (w/v) DSS water. Mice in the WT-DSS+Wy14643 group were treated daily with Wy14643 (2 mg/kg) by i.p. injection, and the other four groups were concurrently given vehicle (1% DMSO sterilized water).

### Cell culture and treatment

AML12 cells (ATCC, cat#CRL-2254) were cultured in DMEM/F12 medium supplemented with 10% fetal bovine serum (FBS), 1% penicillin/streptomycin (P/S), 1% ITS (insulin-transferrin-selenium) supplement, and 40 ng/ml dexamethasone. HepG2 cells (ATCC, cat#HB-8065) were cultured in DMEM/H medium containing 10% FBS and 1% P/S. Mouse primary hepatocytes were obtained from mouse liver by collagenase perfusion and percoll purification, and were cultured in William's E medium containing 10% FBS and 1% P/S. The cells were grown in a 5% CO_2_ humid atmosphere at 37°C.

To investigate the effect of PSM on SCD1 expression *in vitro*, an MTT assay was performed to determine the effects of PSM and CAY 10566 (MCE, Cat#HY-15823) on cell viability of HepG2. Then, the HepG2 cells were treated with 1 µM PSM, 50 µM OA, and 2.5 µM CAY10566 for 24 h before harvest. Mouse primary hepatocytes were treated with 0.5 µM and 1 µM PSM for 24 h, respectively. To verify the inhibition effect of SR9238 on LXRα target genes, HepG2 cells were cultured with DMEM/H containing insulin (10 µg/ml) for 7 d before the treatment of SR9238 (2 µM, 10 µM, and 20 µM) for 24 h. To investigate the effect of CORT on lipid accumulation, AML12 cells were treated with CORT (2 µM, 10 µM, and 50 µM) for 48 h.

### Untargeted metabolomics and lipidomics

Metabolomics and Lipidomics analyses were performed at the Advanced Mass Spectrometry Center of West China Hospital. LC-MS-grade acetonitrile, methanol, isopropanol, and formic acid were obtained from Thermo Fisher Scientific (Waltham, MA), and ammonium acetate was purchased from Macklin (Shanghai, China). The internal standard chlorpropamide was obtained from Sigma-Aldrich (Missouri, USA), and LPC12:0 was bought from Avanti Polar Lipids (Alabaster, AL).

Metabolomics analysis was performed on mice serum and liver samples. Sample preparations were carried out as described previously.^1^ UHPLC-MS analysis was performed using a Vanquish UHPLC-Q-Exactive plus MS system (Thermo Fisher, USA). Chromatographic separation was carried out with an ACQUTY UPLC HSS T3 column (2.1 × 100 mm, 1.8 µm). Mobile phase A was 0.1% formic acid (FA) in water, and mobile phase B was 0.1% FA in acetonitrile (ACN). The flow rate was 0.3 mL/min, with a linear gradient ranging from 2% to 98% ACN over a 17 min run. MS/MS spectra were collected in both positive and negative ion modes and was operated in data-dependent acquisition (DDA) model at m/z 60 to 900. The acquired raw data were processed with MS-DAIL ver.4.38, and SIMCA-P 13.0 (Umetrics, NJ) was used for PCA and OPLS-DA analysis. Targeted analysis was carried out by integrating the peak area of each metabolite using Xcalibur. The list of altered typical metabolites in the serum and liver were present in Tables S1 and S2.

Lipidomic analysis was performed on mice liver samples. Approximately 20 mg of liver tissue was homogenized with a cold methanol/chloroform (1:1) solution containing 5 µM LPC12:0 as an internal standard. Each sample underwent vortexing for 30 s and then centrifuged at 1,2000 rpm for 15 min at 4 °C. The lower organic layer was transferred and dried under vacuum. The residue was resuspended in 25 µL of chloroform/methanol (1:1) solution, followed by diluting with 200 µL of isopropanol (IPA)/ACN/H_2_O (2:1:1). All samples were analyzed using Vanquish UHPLC-Q Exactive plus MS system equipped with an Acquity UPLC CSH C18 column (2.1 × 100 mm, 1.7 µm). For UHPLC, mobile phase A was ACN:H₂O (60:40) with 10 mM ammonium acetate and 0.1% FA, and mobile phase B was IPA: ACN (90:10) with 10 mM ammonium acetate and 0.1% FA. Elution gradient is as follows: 0–2.0 min, 40% B; 2.0–2.1 min, 40%–43%B; 2.1%–12.0min, 43%–50%B; 12.0–12.1 min, 50%–54%B; 12.1–18.0 min, 54%–70%B; 18.0–18.1 min, 70%–99%B; 18.1–20.0 min, 99%–40%B. The flow rate was 0.4 mL/min and the injection volume was 5 µL.

The raw files were processed for lipid annotation, peak extraction and peak alignments using MS-DIAL software (V.4.38). The set of parameters was described as a reference.^33^ All the identified lipids were confirmed by matching their MS/MS spectral fragmentations against the in-built library of MS DIAL. PCA, orthogonal projection to latent structures discriminant analysis (OPLS-DA), and VIP scoring were conducted using MetaboAnalyst 4.0. Significance thresholds were set as fold-change > 2 and adjusted *p* < 0.05 (FDR-corrected). The list of altered typical lipid metabolites was present in Table S3.

## 16S rRNA gene sequencing and data analysis

Total microbial genomic DNA was extracted from the stool samples using the TianGen stool DNA extraction kit (Beijing, China). The bacterial 16S rRNA (V3–V4 regions) was amplified using universal primers 338F/806R and sequenced on the Illumina MiSeq platform. In brief, the raw sequencing data were processed with QIIME 2. Taxonomy was assigned using RDP classifier and annotated with the Silvav138 reference database. Alpha diversity analysis was based on one-way ANOVA followed by Tukey's post hoc test. Principal coordinate analysis (PCoA) plot was used to visualize beta diversity differences based on unweighted UniFrac distances. Kruskal–Wallis and post-hoc Dunn's tests were used for differentially enriched bacteria taxa analysis. The difference of Firmicutes and Bacteroidetes at the phylum level was assessed using student's *t-*test and Mann–Whitney nonparametric test. Bioinformatic analysis was performed and visualized on the online platform of the Majorbio Cloud Platform (Shanghai, China).

### *L. johnsonii* culturing

*L. johnsonii* 6084 was purchased from the China Center of Industrial Culture Collection. (Beijing, China). It was cultured with MRS medium in an anaerobic chamber (Coy) with an atmosphere of 5% hydrogen, 5% carbon dioxide, and 90% nitrogen at 37°C.

For gavaging mice with *L. johnsonii* culture supernatant, *L. johnsonii* was overnight grown to an OD of 1.5 at 600 nm. Then, the bacteria cells and culture supernatant were separated by spinning at 3500 rpm for 10 min. Immediately, 200 µL *L. johnsonii* culture supernatant was gavaged into each mice once every day.

For growth curve and bacterial colony counting, *L. johnsonii* was cultured with 1/2 MRS medium under an initial OD_600_ value of 0.16 and treated with PSM (1 µM, 0.5 µM, and 0.25 µM) for 14 h. The OD_600_ value was measured every two hours using microplate reader. The cell numbers of *L. johnsonii* in the CTRL and 0.5 µM PSM treatment groups were calculated using the flat colony counting method.

### Biochemical assay and immunoenzyme-linked assay

TG, TC in the serum and liver tissue were measured using triglycerides and cholesterol kits (Nanjing Jiancheng Bioengineering Institute, China). The serum LPS were assayed with a mouse LPS ELISA kit (Wuhan Huamei Biotech, China). The levels of interleukin-6 (IL-6) and interleukin-1β (IL-1β) in the serum were detected using Mouse IL-6/IL-1β ELISA kits (Multi Sciences Biotech, China). All the measurements were conducted by following the manufacturer's instructions.

### Histological analysis

For H&E and AB-PAS staining, liver and colon tissues were fixed and embedded in paraffin. 4 µm-thick tissue sections were stained with Hematoxylin and eosin (H&E), and Alcian blue-periodic acid Schiff (AB/PAS), respectively. The histological assessment of inflammation was scored as described in a previous study.^1^ For Masson's trichrome staining, liver tissue was stained using Masson staining kit (Baso Diagnostics Inc., China). For Oil Red O staining, liver tissue and cells were stained following the instructions of Oil Red O staining kit (Baso Diagnostics, China). Liver tissue images were captured within two hours by a microscope (Nikon DS-Ri2, Japan). The cells were visualized by a microscope (Nikon Ti2, Japan).

### QPCR, western blot, immunohistochemistry analysis

The primer sets for RNA analysis are listed in Table S4. Antibodies used in western blot and immunohistochemistry (IHC) are as follows: GAPDH (ET1601-4, Huaan Biotechnology, China, WB 1:1000), TLR2 (R23333, Zen-bioscience, China, WB 1:1000), BSEP (PB9414, Boster Biological Technology, WB 1:1000), SCD1 (2794, Cell Signaling Technology, USA, WB 1:1000), SCD1 (HA723753, Huaan Biotechnology, China, IHC 1:500), LXRα (ET1704-51, Huaan Biotechnology, China, WB 1:1000, IHC 1:500), SREBP1 (HA500210, Huaan Biotechnology, China, WB 1:2000, IHC 1:500), FASN (ET1701-91, Huaan Biotechnology, China, WB 1:2000, IHC 1:500), CD36 (ET1701-24, Huaan Biotechnology, China, WB 1:1000), Fabp4 (ET1703-98, Huaan Biotechnology, China, WB 1:2000), β-actin (C21N23, Selleck Chemicals, USA, WB 1:1000), PPARα (ab126285, abcam, USA, WB 1:1000).

### Statistical analysis

Pearson correlation analysis was performed between differential metabolites and disease phenotype indicators. Data visualization was generated using the OmicStudio tools at http://www.omicstudio.cn/tool. Statistical significance between two groups was assessed using an unpaired two-tailed Student's *t*-test. One-way ANOVA followed by Dunnett's post hoc test was used for multiple group comparisons. Statistical analyses were performed by GraphPad Prism 8.0 (CA, USA). Data were presented as mean ± SD. *, #, *p* < 0.05; **, ##, *p* < 0.01; ***, ###, *p* < 0.001.

## Results

### PSM alleviates DSS-induced experimental UC in mice

In the present study, 3% (w/v) DSS treated mice were used as a model of severe colitis with comorbid liver injury.^34^ To investigate the protective effects of PSM, it was given to mice i.p. once every two days. A schematic diagram was constructed to intuitively describe the animal experiment (Figure 1b). UC manifestations developed in all the mice that had free access to DSS water. PSM-treated colitis mice have relatively stable body weight, reduced DAI score, longer colon, normal-sized spleen, and larger cecum (Figure 1c–h). Histological evaluation showed PSM treatment significantly relieved colonic mucosa damage, inhibited inflammatory cell infiltration, and reduced the loss of goblet cells (Figure 1**i**). In addition, PSM treatment remarkably reduced systemic levels of IL-6 and IL-1β (Figure 1j), and inhibited transcription of colonic IL-6, IL-1β and interleukin-17A (IL-17A) (Figure 1k), whereas upregulated the expression of zonula occludens-1 (ZO-1), occludin and claudin, suggesting that it relieved inflammation and restored impaired intestinal mucosal barrier (Figure 1l).

### PSM alleviates experimental UC-associated liver injury in mice

**Figure 2.**
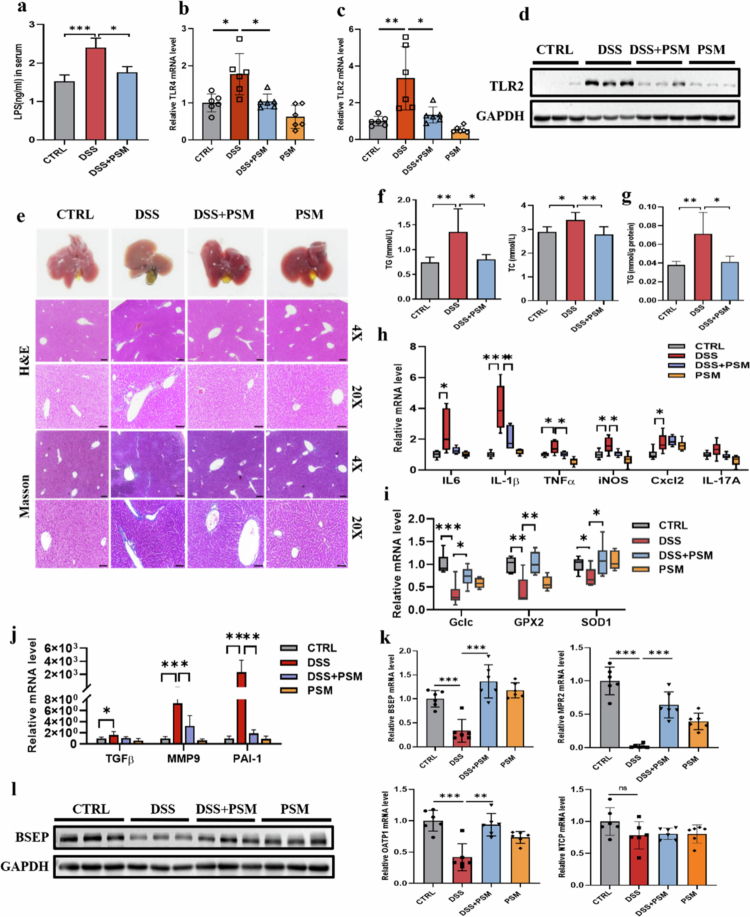
PSM alleviates experimental colitis-associated liver injury. (a) LPS concentration in serum (*n*=5). (b) Relative gene expression of hepatic TLR4 (*n*=6). (c) Relative gene expression of hepatic TLR2 (*n*=6). (d) Hepatic protein level of TLR2 was assessed by western blotting (*n*=3). (e) Representative images of livers, H&E and Masson's trichrome staining of liver sections (scale bar, 100 µm) (*n*=6). H&E staining showed increased inflammatory cell infiltration, Masson's trichrome staining showed increased collagen in the liver tissue. (f) Serum TG and TC concentrations (*n*=6). (g) Liver TG concentration (*n*=6). (h) Relative hepatic gene expression of inflammatory cytokines (*n*=6). (i) Relative expression of anti-oxidative genes (*n*=6). (j) Relative expression of hepatic fibrosis-related genes (*n*=6). (k) Relative gene expression of hepatic bile acid transporters (*n*=6). (l) Hepatic protein level of BSEP was assessed by Western blotting (*n*=3). *p < 0.05, **p < 0.01, ***p < 0.001.

DSS could disrupt the intestinal epithelial barrier and cause bacteria translocation. Increased circulatory LPS and lipoteichoic acid (LTA) triggered proinflammatory signals via TLRs (toll-like receptors), leading to the hepatic inflammatory response and liver damage.^34-36^ PSM treatment significantly decreased DSS-elevated serum LPS and inhibited the transcription of hepatic TLR4 (Figure 2a and b). It also reduced TLR2 expression at both the gene and protein levels (Figure 2c and d). Although no difference of liver size and weight was observed, the liver color shifted to pale, and gallbladders were significantly dilated and filled with dark green bile, implying possible liver steatosis and cholestasis in the comorbid mice. However, treatment with PSM reversed these phenotypic characteristics (Figure 2e). At the biochemical level, it reduced the aberrant total triglyceride (TG) in both serum and liver tissue, along with the increased total serum cholesterol (TC) (Figure 2f and g). At the histological level, it reduced mild inflammation in the liver tissue, which is characterized by vacuolar degeneration of hepatocyte and inflammatory cell infiltration (Figure 2e). Accordingly, PSM treatment significantly inhibited upregulated mRNA levels of inflammatory cytokines (Figure 2h), whereas it increased the expression of downregulated anti-oxidative genes (Figure 2i). Reduced accumulation of collagen in liver tissues following PSM administration was observed (Figure 2e). Consistently, transcription of fibrosis marker genes, including matrix metalloproteinase 9 (MMP) and plasminogen activator inhibitor 1 (PAI-1)(PAI-1), was remarkably suppressed (Figure 2j). Furthermore, PSM treatment alleviated cholestasis, as evidenced by markedly reversing the reduction of BA transporters transcription, including bile salt export pump (BSEP), organic anion transporting polypeptide 1 (Oatp1) and multidrug resistance-associated protein 2 (MRP2), along with the upregulation of BSEP protein level (Figure 2k and l).

### PSM promotes hepatic lipid metabolism

A broad metabolic dysfunction is typically apparent in UC patients.^37^ To investigate the metabolic shifts correlated with host inflammation that were regulated by PSM, untargeted LC‒MS metabolomics was performed. The major classes of identified differential serum metabolites were LPC, Lysophosphatidylethanolamine (LPE) and acylcarnitine (Figure S1a). Interestingly, we found the altered LPC and LPE were all unsaturated, and their abundances were decreased in the DSS group. In contrast, the acylcarnitine levels exhibited an opposite pattern. Notably, treatment with PSM reversed the alterations. The variable importance in projection (VIP) scores showed these unsaturated LPC and LPE drove clustering, with LPC 18:2, LPC18:1 and LPC 20:4 being the top three upregulated serum metabolites by PSM treatment (Figure S1b).

Since the degradation of PC and PE generates LPC, LPE and free fatty acid (FFA), and the effects of specific FFA on UC can vary significantly,^17^ we analyzed the hepatic levels of major FFA. Similarly, we observed that colitis significantly decreased unsaturated FFA, such as linoleic acid and palmitoleic acid, whereas increased saturated FFA, including stearic acid and palmitic acid. Accordingly, the peak ratios of saturated free fatty acid (sFFA) to unsaturated free fatty acid (usFFA) were significantly increased in the DSS group. However, PSM treatment improved the imbalance of FFA and reduced the peak ratios of sFFA to usFFA (Figure S1c and d).

**Figure 3.**
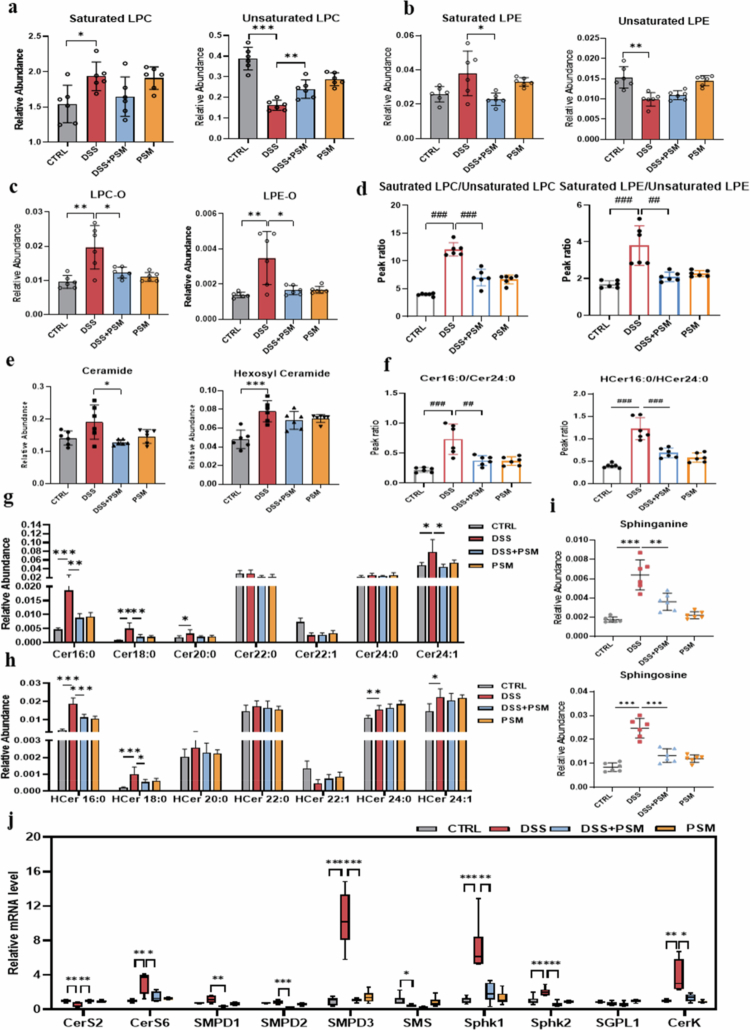
Profiles of hepatic lipidome in mice. (a) Relative abundance of altered total saturated and unsaturated LPC (*n*=6). (b) Relative abundance of altered total saturated and unsaturated LPE (*n*=6). (c) Relative abundance of differential total LPC-O and LPE-O (*n*=6). (d) Peak ratios of altered total saturated LPC/unsaturated LPC and altered total saturated LPE/unsaturated LPE. (e) Relative abundance of differential total ceramide and hexosyl ceramide (*n*=6). (f) Peak ratios of Cer16:0/Cer24:0 and Hcer16:0/Hcer24:0. (g) Relative abundance of ceramide species (*n*=6). (h) Relative abundance of Hexosyl ceramide species (*n*=6). (i) Relative abundance of sphinganine and sphingosine (*n*=6). (j) Relative gene expression of key enzymes involved in ceramide synthesis and degradation (*n*=5). *, #*p* < 0.05, **, ##*p* < 0.01, ***, ###*p* < 0.001.

All these results suggest that PSM significantly improves the hepatic lipid metabolic profile of the comorbid mouse model and that specific lipid species may play crucial roles in the protective effects of PSM. To further investigate this mechanism, we employed untargeted lipidomics for comprehensive profiling of the hepatic lipidome. Lysophospholipids (LPLs) and sphingolipids were identified as the two major lipid classes altered by PSM (Figure 3, Figure S1). Interestingly, the total levels of saturated LPC (sLPC) and LPE (sLPE), along with total Lysophosphatidylcholine-O (LPC-O) and Lysophosphatidylethanolamine-O (LPE-O) were remarkably higher in colitis, whereas total unsaturated LPC (usLPC) and LPE (usLPE) dramatically decreased. PSM treatment significantly reduced total sLPE, LPC-O and LPE-O and increased total usLPC, respectively (Figure 3a–c). Moreover, the ratios of total sLPC to usLPC and sLPE to usLPE were decreased after PSM administration (Figure 3d). As for the sphingolipids, an aberrant accumulation of ceramides (Cers), hexosyl ceramides (Hcers), and sphingomyelins (SMs) was observed. However, PSM reduced the total levels of Cers and SMs (Figures 3e and S1e) and normalized the corresponding ratios of Cer 16:0 to Cer 24:0 and Hcer 16:0 to Hcer 24:0, which are promising biomarkers for metabolic disease (Figure 3f)^38^. Specifically, the species of Cers, Hcers and SMs reduced by PSM treatment were mainly saturated long-chain sphingolipids, including Cer16:0, Cer18:0, Hcer16:0, Hcer18:0, SM16:0, SM18:0, and SM20:0 (Figures 3g and h, S1f). Next, we performed correlation analysis between differential lipids and liver injury indicators. usLPLs other than LPE18:2 exhibited a significant negative correlation with liver injury. In contrast, sLPLs, LPC-O and LPE-O, along with Cers and HCers, were positively associated (Figure S1g). The consistent pattern was also observed between the differential lipids and colon injury (Figure S1h).

Given that ceramide is the central core of sphingolipid metabolism, we further analyzed the precursors and key enzymes for ceramide synthesis and degradation. As expected, abnormally elevated sphinganine and sphingosine were both significantly reduced following PSM treatment (Figure 3i). Accordingly, it effectively suppressed the expression of enzyme-encoding genes, which providing a mechanistic basis for the observed ceramide accumulation in the liver, including ceramide synthase 6 (CerS6), sphingomyelin phosphodiesterase 1/2/3 (SMPD1/2/3), sphingosine kinase 1/2 (Sphk1/2), and ceramide kinase (CerK) (Figure 3j).

### PSM-induced upregulation of hepatic SCD1 involves the gut microbiome

**Figure 4.**
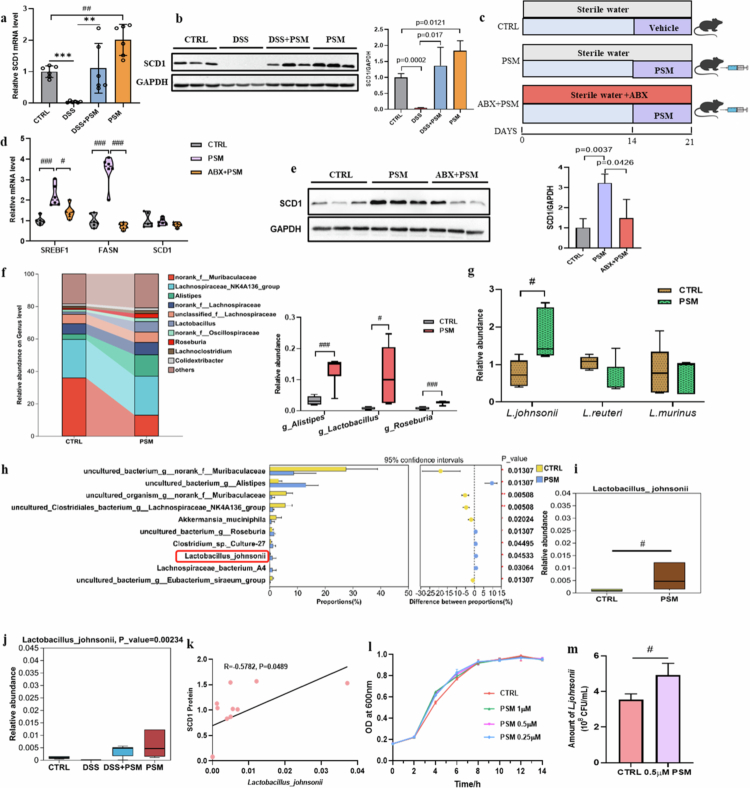
PSM upregulates hepatic SCD1 by enriching *L. johnsonii*. (a) Relative gene expression of hepatic SCD1 (*n*=6). (b) Hepatic protein level of SCD1 was assessed by Western blotting and the densitometric quantification (*n*=3). (c) Schematic diagram of the mouse experiment. (d) Relative expression of hepatic SCD1 and its upstream regulatory genes (*n*=6). (e) Protein level of hepatic SCD1 in the CTRL, PSM alone and ABX+PSM groups, along with densitometric quantification (*n*=3). (f) Relative abundance of altered microbiota by PSM alone at the genus level (*n*=6). (g) Relative abundance of three *Lactobacillus* species in stool samples quantified by QPCR (*n*=5). (h) Differential abundance of the fecal microbiota at the species level (*n*=6). (i) Relative abundance of *L. johnsonii* was increased in the PSM alone group (*n*=6). (j) Relative abundance of *L. johnsonii* in each group (*n*=6). (k) Correlation analysis of the relative abundance of *L. johnsonii* and hepatic SCD1 protein expression. (l) Growth curves of *L. johnsonii*cultured with different concentrations of PSM at the indicated time points (*n*=3). (m) Bacterial colony counts of *L. johnsonii*. *, #*p* < 0.05, *, ##*p* < 0.01, *, ###*p* < 0.001.

SCD1 is a key enzyme in hepatic de novo lipogenesis, responsible for the conversion of SFAs into MUFAs. SCD1 deficiency can result in the reduction of MUFAs, and induces de novo ceramide synthesis, leading to its accumulation.^22^ ^,^ ^39^ ^,^ ^40^ Since PSM treatment increased MUFAs and reduced ceramide accumulation in the liver, we hypothesized that SCD1 may play a role in PSM-remodeled hepatic lipid metabolism. Subsequently, the gene and protein levels of hepatic SCD1 in the mice were analyzed. In line with prior findings,^35^ DSS dramatically suppressed SCD1 at both the transcriptional and translational levels, but the repressions were significantly restored by PSM administration. Surprisingly, compared to the CTRL group, PSM alone significantly induced SCD1 expression, suggesting that it may directly upregulate this enzyme rather than merely reverse DSS-triggered downregulation (Figure 4a and b).

To determine our speculation, we tested PSM *in vitro* on both the AML12 cell line and mouse primary hepatocytes. However, no changes in SCD1 expression were observed (Figure S2a–g). The discrepancy between *in vivo* and *in vitro* effects of PSM on SCD1 suggests that hepatic SCD1 regulation involves entities that are absent *in vitro*, such as gut commensal bacteria. Thus, to better understand whether the upregulation of hepatic SCD1 by PSM is in a microbiota-dependent manner, we depleted the endogenous microbial community in mice by antibiotics (ABX). A schematic diagram was constructed to intuitively describe the animal experiment (Figure 4c). Subsequently, we analyzed the expression of upstream genes of SCD1, which are involved in hepatic de novo lipogenesis. It showed transcriptions of both sterol regulatory element-binding factor-1 (SREBF1) and FASN were enhanced by PSM alone (Figure 4d). Consistent with prior result, the protein expression of hepatic SCD1 was distinctly increased in the PSM alone group. Conversely, a significant reduction was observed between the PSM alone and ABX + PSM groups (Figure 4b and e). Together, these data demonstrated that the gut microbiota is essential for PSM to upregulate hepatic SCD1 in mice.

### PSM enriches the gut commensal *Lactobacillus johnsonii*

To further investigate the effects of PSM on the gut microbiota and identify the specific microbe that may play crucial roles in hepatic SCD1 upregulation, the 16S rRNA sequencing was performed. Interestingly, compared to the CTRL group, the genera of *Alistipes*, *Lactobacillus* and *Roseburia* were significantly enriched in the PSM alone group (Figure 4f). We hence analyzed the most abundant *Lactobacillus* species in normal mice, including *Lactobacillus murinus*, *Lactobacillus reuteri* and *L. johnsonii* by qPCR. In accordance with the 16S rRNA sequencing data, it showed only *L. johnsonii* was markedly enriched by PSM alone (Figure 4g–i). Moreover, the relative abundance of *L. johnsonii* was decreased in colitis and significantly recovered after PSM treatment (Figure 4**j**). Interestingly, a significant positive correlation was observed between the relative abundance of *L. johnsonii* and the hepatic SCD1 protein expression (Figure 4k). Additionally, the *in vitro* study showed that PSM significantly promoted the growth of *L. johnsonii* at concentrations ranging from 0.25 µM to 1 µM, demonstrating it can directly enrich this bacterium (Figure 4l and m).

### LXRα signaling is required for PSM-induced upregulation of hepatic SCD1

Collectively, PSM treatment remodels lipid metabolism via hepatic SCD1 upregulation in a gut microbiota-dependent manner and enriches *L. johnsonii*, whose abundance was positively correlated with hepatic SCD1 expression. Based on these findings, we proposed that *L. johnsonii* served as a pivotal mediator in the PSM-induced upregulation of SCD1, and sought to determine the potential mechanism for SCD1 induction.

A previous study has shown colitis-associated hepatic lipid metabolic disorder is attributed to overall disruption of metabolic processes, including lipogenesis, lipid oxidation and lipolysis.^21^ Hence, we examined whether PSM treatment could promote these processes. Treatment with PSM inhibited FFA uptake by repressing the transcription of cluster of differentiation 36 (CD36), fatty acid-binding protein 4 (Fabp4) and lipoprotein lipase (LPL). Concurrently, it increased the expression of peroxisome proliferator-activated receptor alpha (PPARα) and its target gene acyl-coenzyme A oxidase 1 (Acox1) to promote lipid oxidation. Importantly, it significantly activated hepatic de novo lipogenesis (DNL) by upregulating key lipogenic genes (Figure S3a). Surprisingly, PSM alone induced a significant increase of SCD1, stearoyl-coA desaturase 2 (SCD2) and FASN (Figures 4a, S3b and c), suggesting that its induction on DNL is a direct effect.

Hepatic LXRα activation results in the upregulation of transcription, translation, and proteolytic cleavage of sterol regulatory element-binding protein-1 (SREBP1), a transcription factor that directly regulates FASN, SCD1 and Acaca.^41^ LXRα-SREBP1-SCD1 is a well-established transcriptional pathway that regulates hepatic DNL.^17^ ^,^ ^21^ PSM alone not only upregulated SCD1 but also promoted FASN expression, suggesting that it induced hepatic DNL, and the upregulation of SCD1 is the downstream event of LXRα signaling.

### *Lactobacillus johnsonii* activates hepatic LXRα–SCD1 signaling to ameliorate experimental UC and associated liver injury

**Figure 5.**
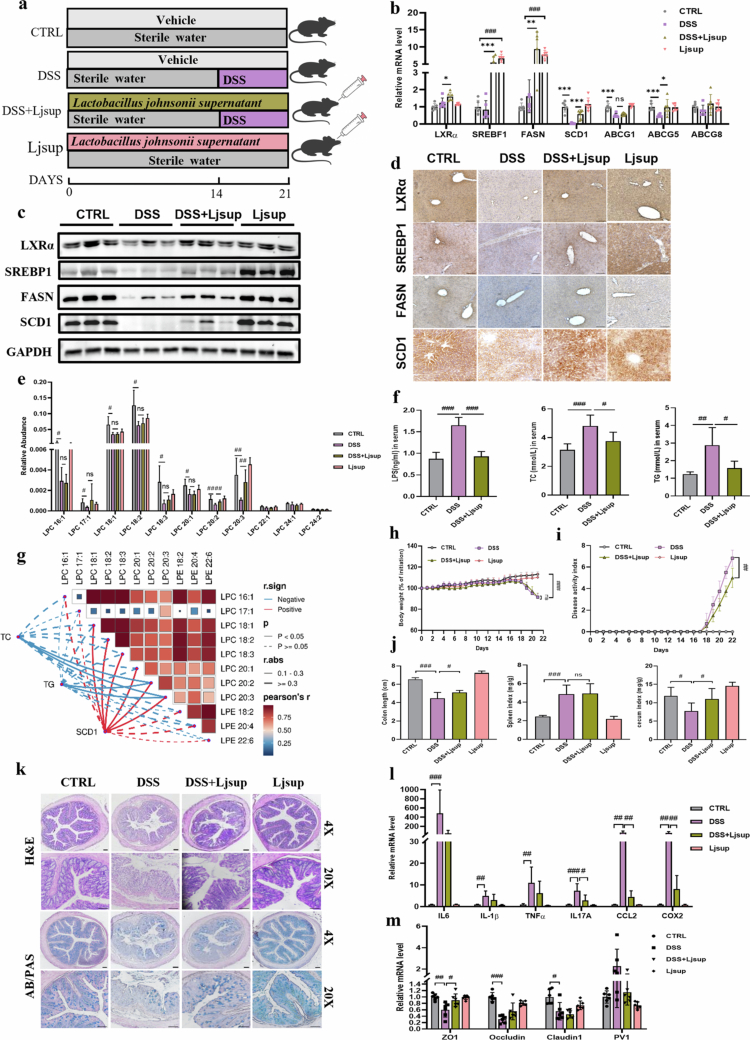
*L. johnsonii* activates hepatic LXRα–SCD1 signaling to ameliorate liver injury and colitis. (a) Schematic diagram of the mouse experiment. (b) Relative expression of LXRα downstream target genes (*n*=6). (c) Levels of LXRα–SCD1 signaling pathway-related proteins in mice liver (*n*=3). (d) Hepatic immunohistochemistry staining showed increased protein expression (LXRα, SREBP1, FASN and SCD1) by culture supernatant of *L. johnsonii* (Ljsup) (*n*=3). (e) Relative abundance of liver LPC species altered by Ljsup (*n*=5–6). (f) Serum LPS, TC and TG concentrations (*n*=6). (g) Correlation analysis of differential LPC and LPE species with the serum TC, TG concentrations and hepatic SCD1 protein expression. (h, i) Body weight changes and disease activity index (DAI) during the experiment (*n*=6). (j) Colon length, spleen and cecum index on day 8 (*n*=6). (k) Representative H&E and AB/PAS staining of distal colon sections (scale bar, 100 µm) (*n*=3). (l) Ljsup supplementation reduced the relative expression of colonic inflammatory cytokines (*n*=6). (m) Ljsup supplementation increased the relative expression of colonic ZO-1 (*n*=6). *, #*p* < 0.05, **, ##*p* < 0.01, ***, ###*p* < 0.001.

To investigate whether *L. johnsonii* was the pivotal mediator of PSM's effect on SCD1 upregulation, mice were treated with culture supernatant of *L. johnsonii* (Ljsup) for two weeks before the comorbid model was induced (Figure 5a). Then, we examined the expression of genes regulating hepatic lipid metabolism (Figure S4a). Ljsup remarkably increased the mRNA levels of LXRα and its downstream target genes in mice with comorbidity. Interestingly, Ljsup alone significantly increased the transcription of SREBF1 and FASN for around 6-fold and 7-fold, respectively (Figure 5b). At the protein level, the hepatic DNL signaling pathway was markedly suppressed, proofed by decreased LXRα, SREBP1, FASN and SCD1. However, Ljsup treatment counteracted this inhibition, and Ljsup alone enhanced SREBP1 expression (Figure 5c, d). These data suggested *L. johnsonii* could activate the hepatic LXRα-SREBP1-SCD1 signaling pathway in a direct way.

As for the lipid profile, Ljsup treatment significantly increased the relative abundance of usLPLs, including LPC20:2, LPC20:3 and LPE20:4, which was confirmed by both liver lipidomics and serum metabolomics data (Figures 5e, S4b and c). We next measured circulatory inflammatory markers and biochemical parameters. Significantly higher levels of LPS, TC and TG were detected in mice serum of the comorbid model group. Treatment with Ljsup reversed the elevation, suggesting that it could effectively relieve systemic inflammation and hepatic lipid disorder (Figure 5f). Furthermore, the relative abundance of LPC20:2 and LPC20:3, which was increased by Ljsup administration was significantly positively correlated with hepatic SCD1 expression, and showed strong negative correlation with serum levels of TC and TG (Figure 5g), indicating an important potential in the liver protective effects of Ljsup.

Next, UC phenotype indices, such as body weight, DAI score, colon length, the spleen and cecum index, were analyzed. As expected, Ljsup treatment significantly reduced the DAI score, whereas increased the colon length and cecum index (Figure 5h-j). Colon tissue section staining showed mild inflammatory cell infiltration and less loss of goblet cells. Consistently, the expressions of key mediators of inflammation, such as IL17A, C-C Motif Chemokine Ligand 2 (CCL2) and Cyclooxygenase-2 (COX2) were reduced, whereas tight junction molecule ZO-1 transcription was enhanced, suggesting the inhibition of inflammation and recovery of intestinal epithelial barrier following Ljsup administration (Figure 5k). Similarly, LPC20:2 and LPC20:3 exhibited significant negative correlations with DAI score, but strongly positively correlated with colon length (Figure S4d), implying a beneficial effect on colitis. Together, these results support the idea that Ljsup promotes the production of LPC20:2 and LPC20:3 via activating LXRα-SREBP1C-SCD1 pathway to remodel hepatic lipid metabolism.

### SR9238 abolishes the liver protection and anti-colitis effects of *Lactobacillus johnsonii* in comorbidity

**Figure 6.**
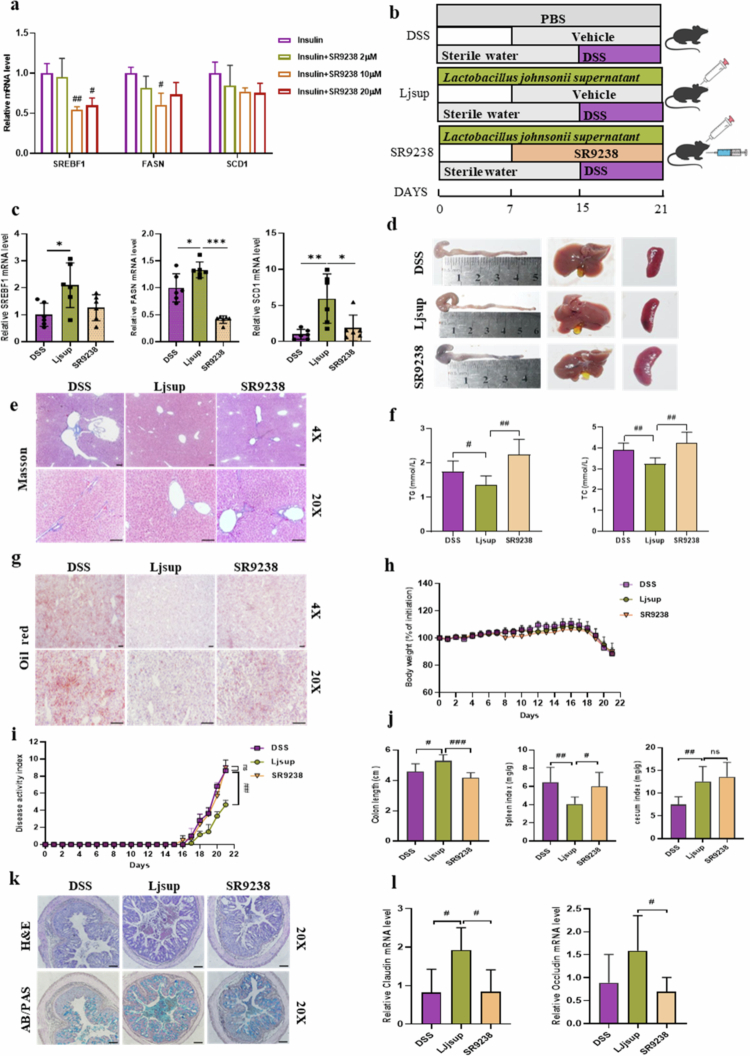
SR9238 abolishes the liver protection and anti-colitis effects of *L. johnsonii*. (a) Relative mRNA levels of SREBF1, FASN and SCD1 in HepG2 cells pretreated with insulin (10 µg/ml) for 7 d, followed by 2 µM, 10 µM, and 20 µM SR9238 treatment (*n*=3). (b) Schematic diagram of the mouse experiment. (c) Relative expression levels of hepatic SREBF1, FASN and SCD1 in mice (*n*=6). (d) Representative images of mice colon, liver and spleen. (e) Masson's trichrome staining showed increased collagen in liver sections (scale bar, 100 µm) (*n*=3). (f) Serum TG and TC concentrations (*n*=6). (g) Oil Red O staining showed incereased lipid droplet accumulation (scale bar, 100 µm) (*n*=3). (h, i) Body weight changes and disease activity index (DAI) changes during the experiment (*n*=6). (j) Colon length, spleen and cecum index (*n*=6). (k) Representative H&E and AB/PAS staining of distal colon sections (scale bar, 100 µm) (*n*=3). (l) SR9238 reduced the relative expression of claudin and occludin in colon tissue (*n*=5). *, #*p* < 0.05, **, ##*p* < 0.01, ***, ###*p* < 0.001.

To determine the dependency of *L. johnsonii* on hepatic DNL in alleviating the comorbidity of UC and livery injury, the LXRα inverse agonist SR9238 was used for LXRα signaling inhibition. First of all, the efficacy of SR9238 in suppressing lipogenesis was evaluated *in vitro* ^42^ (Figure 6a). At a concentration of 10 µM, SR9238 significantly inhibited the insulin-induced expression of SREBF1 and FASN on HepG2 cells. Then, we sought to determine whether SR9238 could abolish both the liver protection and anti-colitis effects of *L. johnsonii* by blocking hepatic DNL. A schematic diagram was constructed to intuitively describe the animal experiment (Figure 6b). Consistent with the *in vitro* data, we found that SR9238 treatment resulted in substantial repression of Ljsup-induced lipogenic genes expression (Figure 6c). Compared to the Ljsup group, a distinct appearance of the liver was observed in SR9238 treated mice (Figure 6d). Although the liver size was normal, it became fibrotic with a firm texture and a slightly granular surface. Increased collagen deposition in the liver tissues was confirmed by Masson's trichrome staining (Figure 6e). Also, an exacerbation on hepatic lipid accumulation was confirmed by elevated serum TG and TC levels and Oil Red O staining (Figure 6f and g).

Similarly, although SR9238 treatment did not further reduce body weight (Figure 6h), it leaded to an increased colitis DAI score (Figure 6i), along with colon shortening and spleen enlargement (Figure 6d and j). Histological assessments of colon tissue revealed that SR9238 aggravated intestinal epithelial destruction, inflammatory cell infiltration, and the loss of goblet cells (Figure 6k). Moreover, the expressions of the tight junction molecules claudin and occludin were significantly reduced by SR9238 (Figure 6I). Overall, these results demonstrated that the protective effects of *L. johnsonii* against UC and concomitant liver injury were abolished by SR9238-mediated suppression of LXRα-dependent hepatic DNL.

### CORT aggravates experimental UC with liver injury through the LXRα–SCD1 axis

Since we have revealed the hepatic LXRα‒SCD1 axis plays a crucial role in the *L. johnsonii* mediated protective effects of PSM on UC and concomitant liver injury, we hypothesized that the inhibition of this axis may be a potential pathological mechanism of comorbidity. Interestingly, we found that key metabolites of glucocorticoid metabolism, including corticosterone (CORT), dihydrocorticosterone and 11-dehydrocorticosterone, were abnormally elevated in mice with comorbidities and were significantly reduced following both PSM and Ljsup administrations (Figure S5a). CORT is the most abundant and bioactive endogenic glucocorticoid in rodent. It was confirmed in liver and serum samples by an authentic standard (Figure S5b and c). LXRα plays a crucial role in the regulation of glucocorticoid production.^43^ The impacts of CORT on lipid metabolism and the potential interactions between CORT and LXRα have not been investigated in the mouse model of UC with comorbid liver injury.

**Figure 7.**
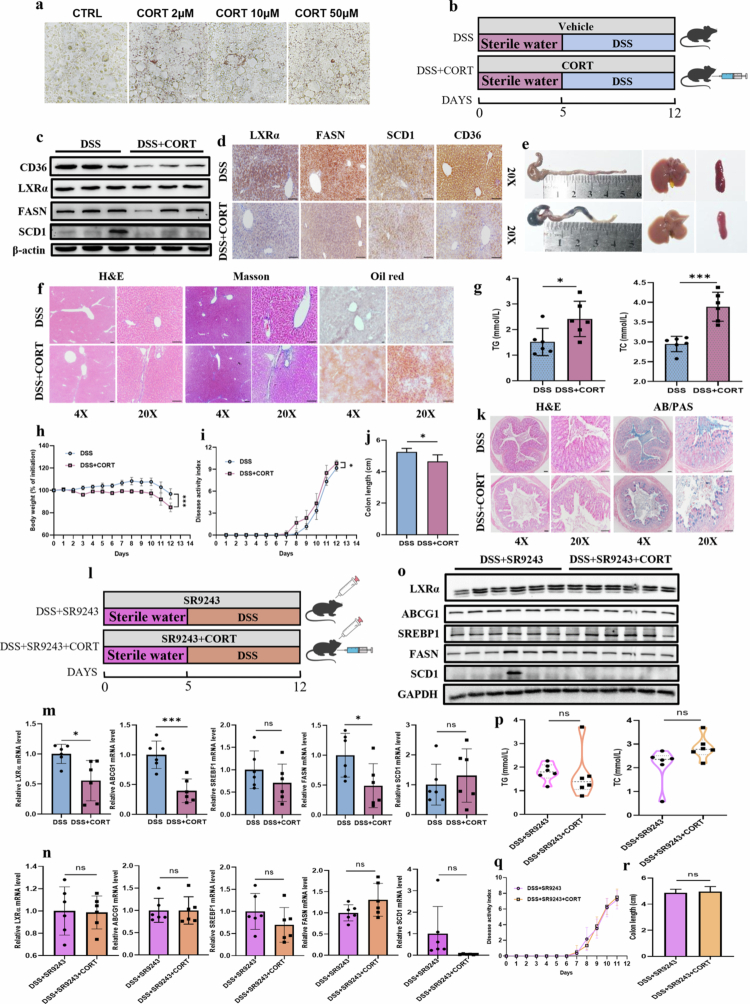
CORT aggravates colitis with liver injury through the LXRα‒SCD1 axis. (a) Oil Red O staining of AML12 cells treated with 2 µM, 10 µM, and 50 µM CORT. (b) Schematic diagram of the mouse experiment. (c) Protein levels of hepatic CD36, LXRα, FASN, and SCD1 in mice. (d) Representative immunohistochemical staining revealed the decreased protein expression (LXRα, FASN, SCD1, and CD36) in mice (*n*=3). (e) Representative images of mice colon, liver and spleen. (f) Representative H&E, Masson's trichrome and Oil Red staining of liver sections (scale bar, 100 µm) (*n*=3). (g) Serum TG and TC concentrations (*n*=6). (h, i) Body weight changes and disease activity index (DAI) during the experiment (*n*=6). (j) CORT reduced colon length (*n*=6). (k) Representative H&E and AB/PAS staining of distal colon sections (scale bar, 100 µm) (*n*=3). (l) Schematic diagram of the mouse experiment. (m, n) Relative mRNA levels of LXRα target genes. (o) Levels of LXRα signaling pathway-related proteins in mouse liver. (p) Serum TG and TC concentrations (*n*=6). (q) Disease activity index (DAI) changes during the experiment (*n*=6). (r) Colon length on day 12. *p < 0.05, **p < 0.01, ***p < 0.001. ns, not significant (*p* > 0.05).

To test if CORT could disrupt lipid metabolism, we treated AML12 cells with CORT for 48 h. Significantly accumulated lipid droplets were observed in the cells (Figure 7a). To determine its role in the comorbid mouse model, the mice were pretreated with CORT for 5 d before inducing comorbidity with DSS. A schematic diagram was constructed to intuitively describe the animal experiment (Figure 7b). Compared with the comorbid model group, CORT treatment further reduced the protein levels of hepatic CD36, LXRα, FASN and SCD1, suggesting the downregulation of LXRα signaling (Figure 7c and d). Additionally, we observed that the liver color turned pale yellow following CORT administration, suggesting more severe hepatic steatosis (Figure 7e), which was confirmed by increased lipid droplets in the cells and elevated TG and TC levels in the serum (Figure 7f, g). Moreover, CORT also exacerbated liver inflammation and fibrosis (Figure 7f). Concurrently, CORT treatment significantly reduced body weight, whereas it increased colitis DAI scores (Figure 7h and i). It also further shortened the colon length, disrupted intestinal epithelial barrier, increased inflammatory cell infiltration, and reduced the number of goblet cells (Figure 7j and k). Together, these data suggest that CORT-induced disruption of the LXRα–SCD1 pathway may aggravate the comorbidity. To confirm that the pathological effects of CORT are through acting on downregulating LXRα signaling, we co-administered CORT with the LXRα antagonist SR9243, which was given daily at a dose that fully suppressed LXRα activity^44^ ^,^ ^45^ (Figure 7I). In line with our hypothesis, in the presence of SR9243, CORT neither downregulated the expression of LXRα-mediated target genes and proteins nor further aggravated the comorbidity (Figure 7m–r), indicating that its pathogenic effects are dependent on LXRα signaling.

### Similar disrupted lipid profile in UC patients

**Figure 8.**
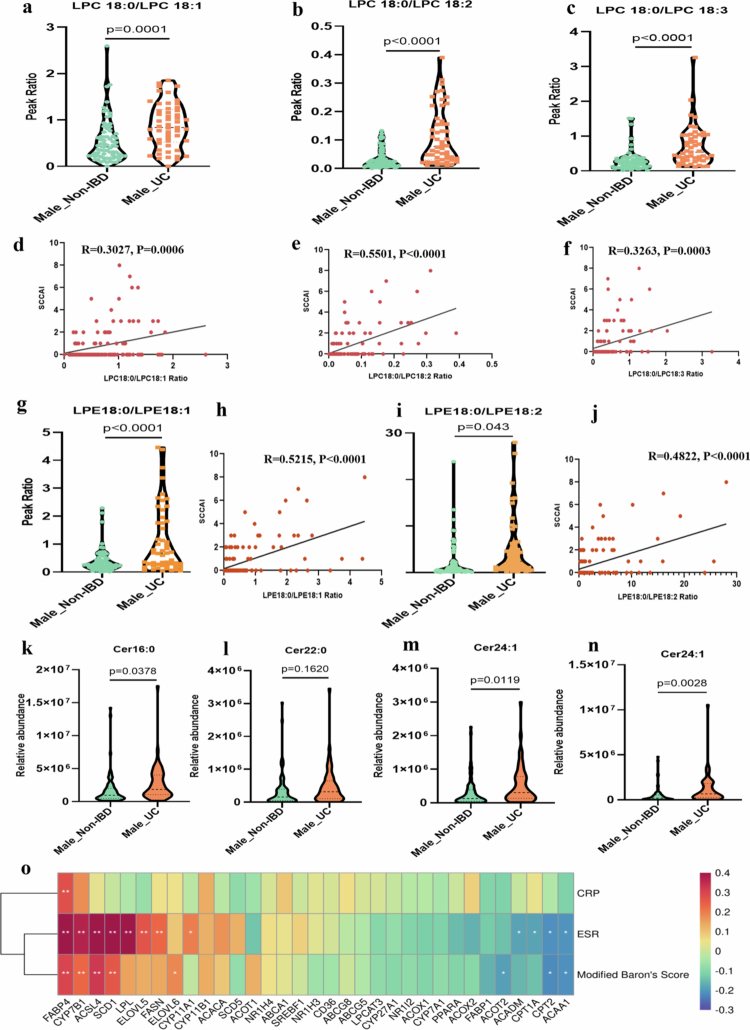
Lipid profile disruption in UC patients. (a–c) Ratios of LPC18:0/LPC18:1, LPC18:0/LPC18:2 and LPC18:0/LPC18:3 (Male_Non-IBD: *n*=73, Male_UC: *n*=53). (d–f) Correlation analysis of the ratios of LPC18:0/LPC18:1, LPC18:0/LPC18:2 and LPC18:0/LPC18:3 with the simple clinical colitis activity index (SCCAI). (g) Ratio of LPE18:0/LPE18:1 (Male_Non-IBD: *n*=73, Male_UC: *n*=53). (h) Correlation analysis of the ratio of LPE18:0/LPE18:1 with the SCCAI. (i) Ratio of LPE18:0/LPE18:2 (Male_Non-IBD: *n*=73, Male_UC: *n*=53). (j) Correlation analysis of the ratio of LPE18:0/LPE18:2 with the SCCAI. (k–n) Levels of ceramide species in stool samples. (o) Correlation analysis of the expression of genes involved in lipid metabolism with UC clinical symptom indices. *p < 0.05, **p < 0.01, ***p < 0.001.

To conﬁrm the clinical relevance of our ﬁndings, we analyzed publicly available data from the project “Longitudinal Metabolomics of the Human Microbiome in Inflammatory Bowel Disease” (metabolomic workbench, study ID: ST000923).^46^ Sex-stratified analyses were performed. The metabolomic profile revealed that the ratios of LPC18:0 to LPC 18:1, LPC 18:0 to LPC 18:2, and LPC18:0 to LPC18:3 significantly increased in male UC patients. Moderate-to-strong positive correlations were observed between these ratios and the simple clinical colitis activity index (SCCAI) (Figure 8a–f). Similarly, the ratios of LPE18:0 to LPE18:1 and LPE 18:0 to LPE18:2 increased and positively associated with the SCCAI (Figure 8g–j). In addition, the levels of ceramide including Cer16:0, Cer24:0 and Cer24:1 significantly elevated (Figure 8o–r). The lipid metabolic alterations were less pronounced in female UC patients, but the pattern of change is similar (Figure 6Sa–i). Consistently, analysis of a public dataset (study ID: ST001000) from another clinical study, “Gut microbiome structure and metabolic activity in inflammatory bowel disease”,^37^ demonstrated that the ratios of sLPC to usLPC were also significantly increased in UC patients compared to the control group (Figure S6j–l). Together, these data robustly support our preclinical findings, suggesting that SCD1 deficiency contributes to lipid dysregulation in UC patients.

To further validate our findings, we retrieved human transcriptomics data from the Human Microbiome Project (HMP2)^46^ and conducted a correlation analysis between the expression of genes related to lipid metabolism and the UC clinical symptom index. The transcription of most genes involved in hepatic lipid synthesis, such as FASN, SCD1 and ELOVL5/6 were highly positively correlated with the erythrocyte sedimentation rate (ESR), whereas SCD1 and ELOVL 6 also positively correlated with the modified Baron's score. By contrast, a strong negative association between the expression of genes involved in lipid oxidation and the clinical symptom index was observed (Figure 8). Collectively, these data suggest that hepatic lipid metabolism, particularly lipogenesis and lipid oxidation, is highly correlated to the disease severity of human UC and may play a critical role in its progression and the occurrence of extraintestinal hepatobiliary diseases. Also, these results provide evidence supporting the potential of SCD1 as a therapeutic target for the clinical management of UC and associated liver injury.

## Discussion

In this study, we demonstrated the protective effects of the natural compound PSM against UC and associated liver injury in mice, revealing that *L. johnsonii* is the pivotal microbe mediating PSM's effects by activating the hepatic LXRα signaling pathway to upregulate SCD1 expression. We also found that abnormally elevated circulating endogenous CORT induced the downregulation of LXRα–SCD1 signaling, and its level was significantly reduced following both PSM and *L. johnsonii* administration, implying that the beneficial effects of PSM and *L. johnsonii* may be partially mediated by improving both systemic CORT circulation and the downstream LXRα–SCD1 signaling axis. These findings suggest that an impaired LXRα–SCD1 axis may contribute to the pathogenesis and progression of UC with liver injury, and PSM, along with *L. johnsonii*, holds promise as novel therapeutic strategies.

UC is associated with alterations in lipid metabolism.^20^ In this study, we found that UC patients exhibited significantly reduced usLPLs and higher levels of ceramide, as well as the expression of genes related to lipid synthesis and oxidation was highly correlated with UC clinical symptom indices. These data indicate that SCD1 and peroxisome-proliferator-activated receptor α (PPARα), two hub genes involved in lipid synthesis and oxidation, respectively, may play crucial roles in the disruption of lipid metabolism in UC. However, their roles remain unclear, and inconsistent findings have been reported in previous studies. While several investigations have demonstrated the therapeutic potential of SCD1,^17^ ^,^ ^47^ one study indicated that its deficiency did not promote DSS-induced colitis.^48^ Similarly, both PPARα activation and suppression have been reported to exert anti-inflammatory effects and alleviate UC.^49^ ^,^ ^50^ In the current study, through a parallel comparative experiment using a specific PPARα agonist (Wy14643) and PPARα knockout mice, we found that PPARα deficiency alleviated colitis in mice, whereas Wy14643 administration did not show a significant protective effect. Interestingly, in DSS-induced colitis, remarkably higher expression levels of hepatic SCD1 were observed in PPARα-deficient mice compared to the PPARα wild-type mice, implying that SCD1 may exert a protective effect against colitis (Figure S7). Moreover, SREBF1 is a transcription factor that controls lipogenesis and is recognized as a regulator of SCD1. One previous study demonstrated that DSS reduced hepatic SREBF1 expression but presumed that this was a downstream event of SCD1 inhibition.^17^ Our findings indicated that DSS disrupted LXRα-mediated de novo lipogenesis in the liver, and LXRα target genes involved in lipogenesis, including SREBF1, FASN and SCD1, are all significantly downregulated, suggesting that SCD1 inhibition is a downstream event of LXRα and SREBF1.

Upregulation of SCD1 by both PSM and *L. johnsonii* treatment increased the levels of usLPCs and usLPEs. The effects of LPC and LPE species on the immune system and inflammation depend on their acyl chain length and degree of saturation.^51^ While the pro-inflammatory effects of sLPC have been widely reported, usLPC, such as LPC18:1, LPC18:2 and LPC20:4, are considered anti-inflammatory lipids and have attracted growing attention. In the present study, we found that liver LPC20:2 and LPC20:3 levels were dramatically elevated upon PSM and *L. johnsonii* administration, and that their levels were significantly negatively associated with both colitis and liver injury. LPC20:2 can undergo enzymatic hydrolysis to release eicosadienoic acid (EDA). Serum levels of EDA are significantly lower in UC patients,^52^ and a recent study demonstrated that oral administration of EDA significantly attenuated UC in mice.^53^ Similarly, increased LPC20:3 may exert protective effects against colitis by serving as a precursor of dihomo-γ-linolenic acid (DGLA, 20:3n-6), which is further converted into anti-inflammatory eicosanoids such as prostaglandin E1.^54^ Together, these usLPC species may contribute to the modulation of gut inflammation and attenuation of UC; however, further studies are required to validate the underlying mechanism.

Cortisol levels in UC patients are dysregulated, and serum cortisol has been suggested as a potential marker of gut‒brain axis interactions in UC patients with comorbid depression.^55^ However, alteration of glucocorticoid metabolism has not been investigated in the context of UC with liver disease. The identification of elevated CORT in our mouse study highlights a previously underexplored metabolic feature of this comorbidity. Importantly, we found that excessive CORT exerted its effects by downregulating the expression of LXRα and its target genes, providing a rationale for future investigations into hypothalamic‒pituitary‒adrenal (HPA) axis dysfunction as a potential contributor to gut‒liver axis pathology. Moreover, our findings suggest that caution may be warranted when using corticosteroids in UC patients with concomitant liver injury.

Several limitations of this work should be noted. First, although the natural compound PSM exhibited various biological activities,^31^ ^,^ ^56^ its development potential as a drug candidate is limited due to its intrinsic properties, particularly the narrow therapeutic window that increases the risk of adverse effects.^57^ However, our study showed that *L. johnsonii* mediated the therapeutic effects of PSM in UC complicated with liver injury, suggesting that this bacterium may represent a promising alternative therapeutic strategy. Second, although we provided substantial evidence that Ljsup administration activated the hepatic LXRα‒SCD1 axis, it remains unclear whether *L. johnsonii* produces LXRα agonists or acts indirectly via host metabolism. Therefore, the precise mechanism underlying LXRα activation requires further investigation. Given the limited evidence that *Lactobacillus* directly produces sterols or other lipid metabolites, LXRα activation is more likely mediated by host metabolic alterations or through interactions with other cholesterol-transforming gut microbes. Last, the UC patient data were obtained from public databases, and the cohorts were not stratified by liver involvement, which may explain the weak correlations between some lipid alterations and the SCCAI. Future studies should validate these findings in independent cohorts and assess additional clinical indices, such as UC Mayo scores and endoscopic scores. Additionally, the weaker statistical significance observed in females may be attributable to sex-related differences in lipid metabolism, potentially influenced by hormonal factors, and warrants further validation in larger cohorts.

In summary, our investigation suggests that the crosstalk between colitis and extraintestinal liver injury is mediated by an extended inflammatory response and elevated circulating endogenous CORT, which induces the downregulation of hepatic LXRα–SCD1 signaling, thereby causing lipid dysregulation and further amplifying colonic inflammation. The natural compound PSM alleviates UC and associated liver injury by enriching *L. johnsonii* to remodel hepatic lipid metabolism, which activates the hepatic LXRα–SCD1 signaling pathway and increases the level of potential anti-inflammation lipid species, namely, LPC20:2 and LPC20:3. Conclusively, our findings provide new insights into the mechanistic exploration of systemic diseases and therapeutic strategies for multi-organ comorbidities.

## Supplementary Material

Supplementary Material Supplementary files (261473828).docx

## Data Availability

The 16S rRNA sequencing raw data have been deposited in the Sequence Read Archive (SRA) with the accession number is PRJNA1397457. The untargeted metabolomics and lipidomics raw data have been deposited to the ProteomeXchange Consortium (https://proteomecentral.proteomexchange.org) (project: IPX0014994000) via the iProX partner repository with the data set identifier PXD072636.
