# Supplementary material for: Lactobacillus johnsonii mediates the protective effects of pristimerin against ulcerative colitis and concomitant liver injury through remodeling hepatic lipid metabolism via LXRα–SCD1 axis
Source: Gut Microbes. 2026 Jul 17;18(1):2701349. doi: 10.1080/19490976.2026.2701349 (PMC13387113; doi:10.1080/19490976.2026.2701349)
Supplement: Supplementary Material — Supplementary files (261473828).docx [file KGMI_A_2701349_SM9333.docx]

**Supplementary information**

***Lactobacillus johnsonii* mediates the protective effects of pristimerin against ulcerative colitis and concomitant liver injury through remodeling hepatic lipid metabolism via LXRα-SCD1 axis**

Yan Cheng^1,2^, Zhanxuan E. Wu^1^, Ruoyue Huang^1^, Qingmei Li^1^, Dongmei Yan^3^, Yuqi Chen^1^, Wei-Feng Zhu^2,4*^, and Fei Li^1*^

^1^ Department of Gastroenterology & Hepatology, Laboratory of Hepato-intestinal Diseases and Metabolism, Frontiers Science Center for Disease-related Molecular Network, West China Hospital, Sichuan University, Chengdu, 610041, China

^2^ School of Pharmacy, Jiangxi University of Chinese Medicine, Nanchang 330004, China

^3^ Academician Workstation, Jiangxi University of Chinese Medicine, Nanchang 330004, China

^4^ Key Laboratory of Modern Preparation of Traditional Chinese Medicine, Jiangxi University of Chinese Medicine, Nanchang, 330004, China

**Figure S1│**Profiles of mice hepatic metabolome and lipidome

**Figure S2│**PSM exerts no effect on SCD1 expression in vitro

**Figure S3│**PSM remodels hepatic lipid metabolism by promoting de novo lipogenesis (DNL)

**Figure S4│**Profiles of hepatic lipid gene expression, lipidome and the serum metabolome altered by *L.johnsonii*

**Figure S5│**PSM and Ljsup treatments restore glucocorticoid metabolism

**Figure S6│**Lipid profile disruption in UC patients

**Figure S7│**PPARα KO-induced upregulation of hepatic SCD1 expression exerts potential benefits for DSS-induced colitis

**Table S1│**Summary of typical altered metabolites by PSM in Serum

**Table S2│**Summary of typical altered metabolites by PSM in liver

**Table S3│**Summary of typical altered lipids by PSM in liver

**Table S4│**Sequences of real time PCR primers

**
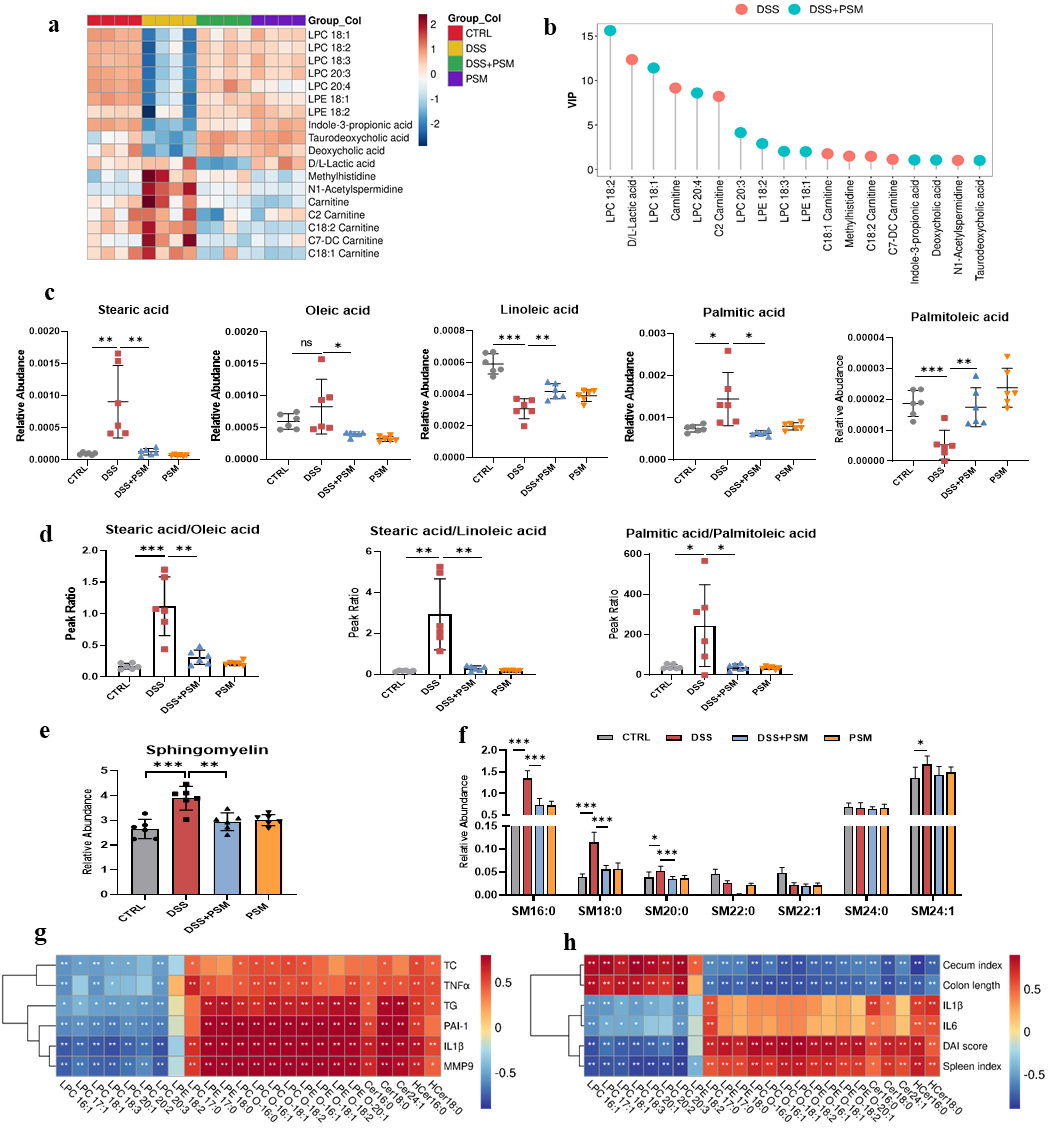
**

**Figure S1 (Related to Figure 3). Profiles of mice hepatic metabolome and lipidome. a** Heatmap of differential metabolites in serum of mice (n=6). **b** VIP score plot of lipid metabolites in serum of paired colitis mice and PSM treated colitis mice. **c** Relative abundance of SA, OA, LA, PA and POA in livers (n=6). **d** Peak ratios of SA to OA, SA to LA and PA to POA (n=6). **e** Relative abundance of total sphingomyelin (SM) in liver.

Heatmap of liver differential lysophospholipids (n=5). **f** Relative abundance of liver SM species. **g, h** Pearson correlation analysis of differential liver lipid metabolites with liver injury indicators, and colon health and injury indicators. **p* < 0.05, ***p* < 0.01, ****p* < 0.001.


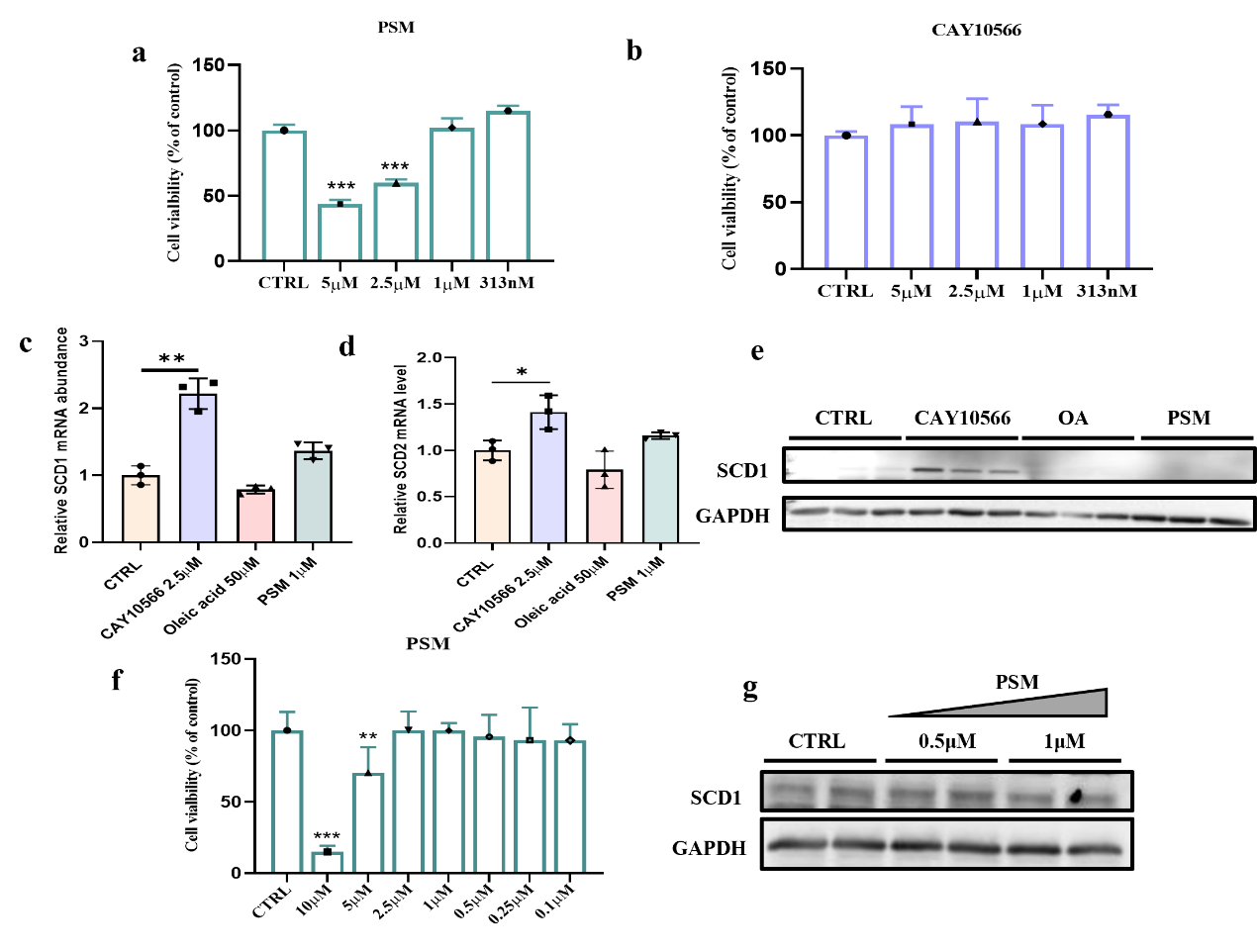


**Figure S2** **(Related to Figure 4). PSM exerts no effect on SCD1 expression *in vitro*. a, b** Influence of PSM on cell viability of AML12 (n=6). **c, d** Relative gene expression of SCD1 and SCD2 in AML12 cells treated with 2.5 µM CAY10566, 50 µM OA and 1 µM PSM (n=3). **e** Protein level of SCD1 in AML12 cells treated with 2.5 µM CAY10566, 50 µM OA and 1 µM PSM (n=3). **f** Influence of PSM on cell viability of mice primary hepatocytes (n=6). **g** Protein level of SCD1 in mice primary hepatocytes treated with 0.5 µM and 1 µM PSM (n=2). **p* < 0.05, ***p* < 0.01, ****p* < 0.001.


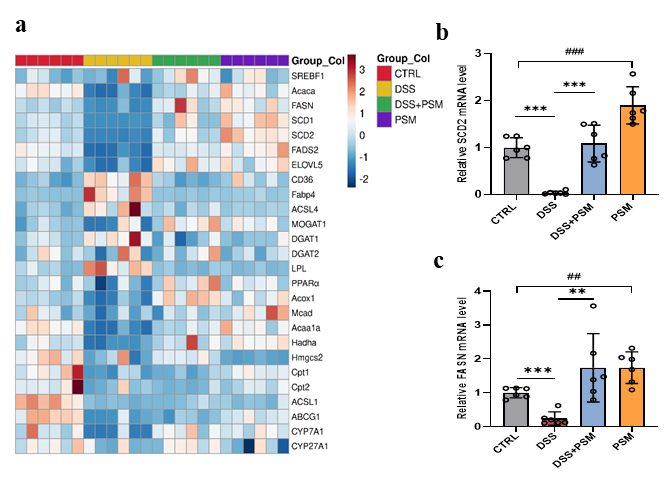


**Figure S3** **(Related to Figure 4). PSM remodels hepatic lipid metabolism by promoting de novo lipogenesis (DNL). a** Heatmap of hepatic lipid gene expression (n=6). **b,c** Relative expression of SCD2 and FASN (n=6). *, #*p* < 0.05, **, ##*p* < 0.01, ***, ###*p* < 0.001.


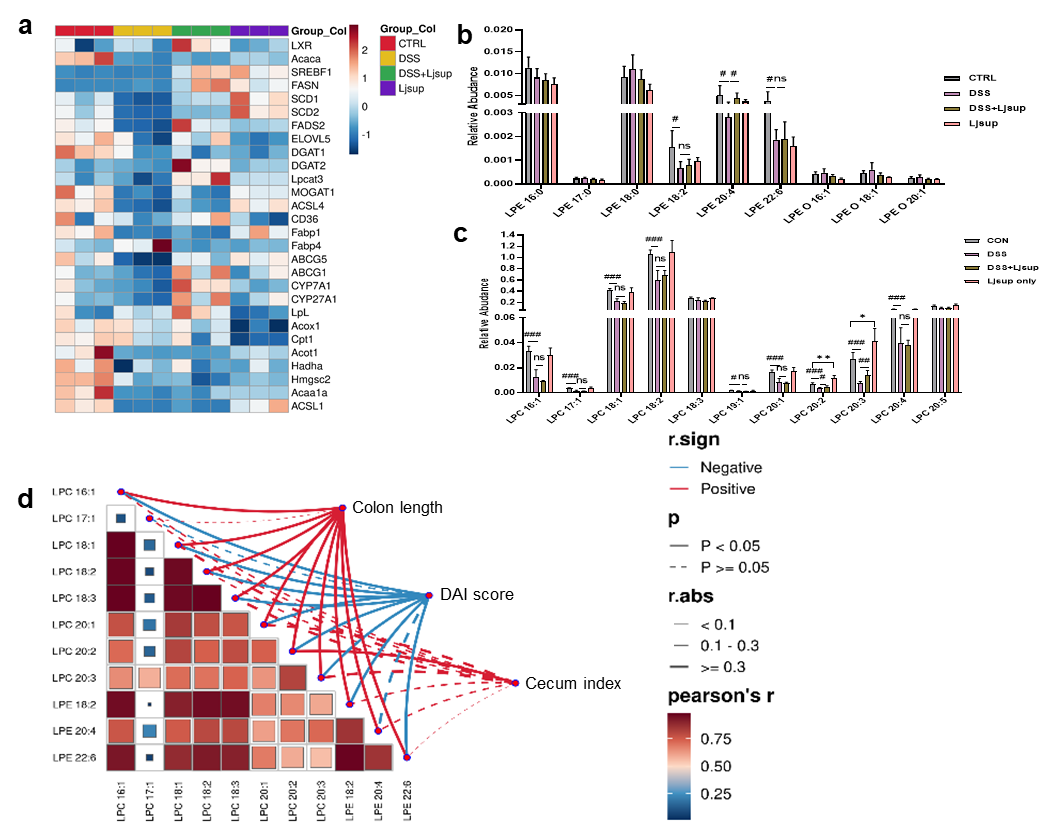


**Figure S4 (Related to Figure 5) Profiles of the hepatic lipid gene expression, hepatic lipidome and serum metabolome that altered by *L.johnsonii*.** **a** Heatmap of hepatic lipid gene expression (n=3). **b** Relative abundance of LPE species in liver altered by culture supernatant of *L.johnsonii* (Ljsup) (n=5). **c** Relative abundance of serum LPC species altered by Ljsup (n=5). **d** Pearson correlation analysis differential LPC and LPE species with colitis phenotype indicators. *, #*p* < 0.05, **, ##*p* < 0.01, ***, ###*p* < 0.001.

**
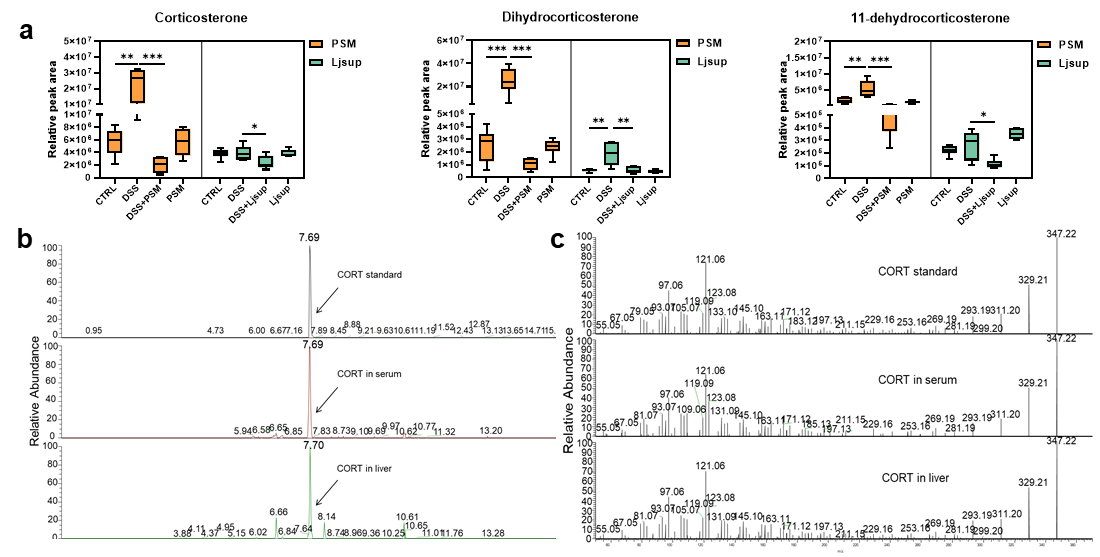
**

**Figure S5 (Related to Figure 7).** **PSM and Ljsup treatments restore glucocorticoid metabolism. a** PSM and Ljsup reduced the elevated corticosterone (CORT), dihydrocorticosterone and 11-dehydrocorticosterone that accumulated in liver and serum of mice with comorbidity, respectively (n=6). **b** Chromatograms of CORT obtained from standard and samples. **c** MS fragments of CORT obtained from standard and samples. **p* < 0.05, ***p* < 0.01, ****p* < 0.001.


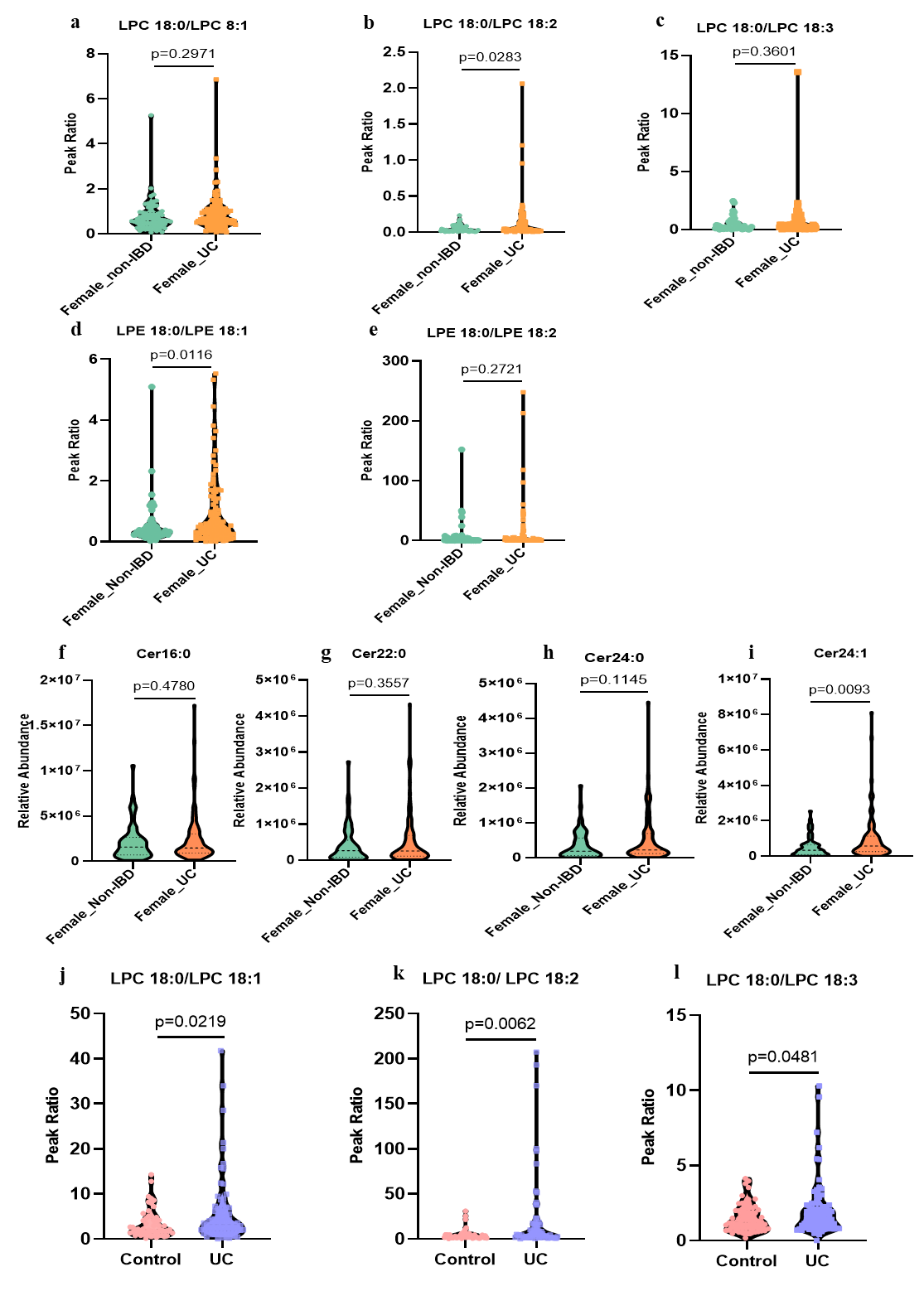


**Figure S6** **(Related to Figure 8). Lipid profile disruption in UC patients. a-c** Ratios of LPC18:0/LPC18:1, LPC18:0/LPC18:2 and LPC18:0/LPC18:3 (Female_Non-IBD: n=61, Female_UC: n=93). **d, e** Ratio of LPE18:0/LPE18:1 (Female_Non-IBD: n=61, Female_UC: n=93). **f-i** Level of ceramide species in stool samples (Female_Non-IBD: n=61, Female_UC: n=93). **j-l** Ratios of LPC18:0/LPC18:1, LPC18:0/LPC18:2 and LPC18:0/LPC18:3 (Control: n=56, UC: n=76).


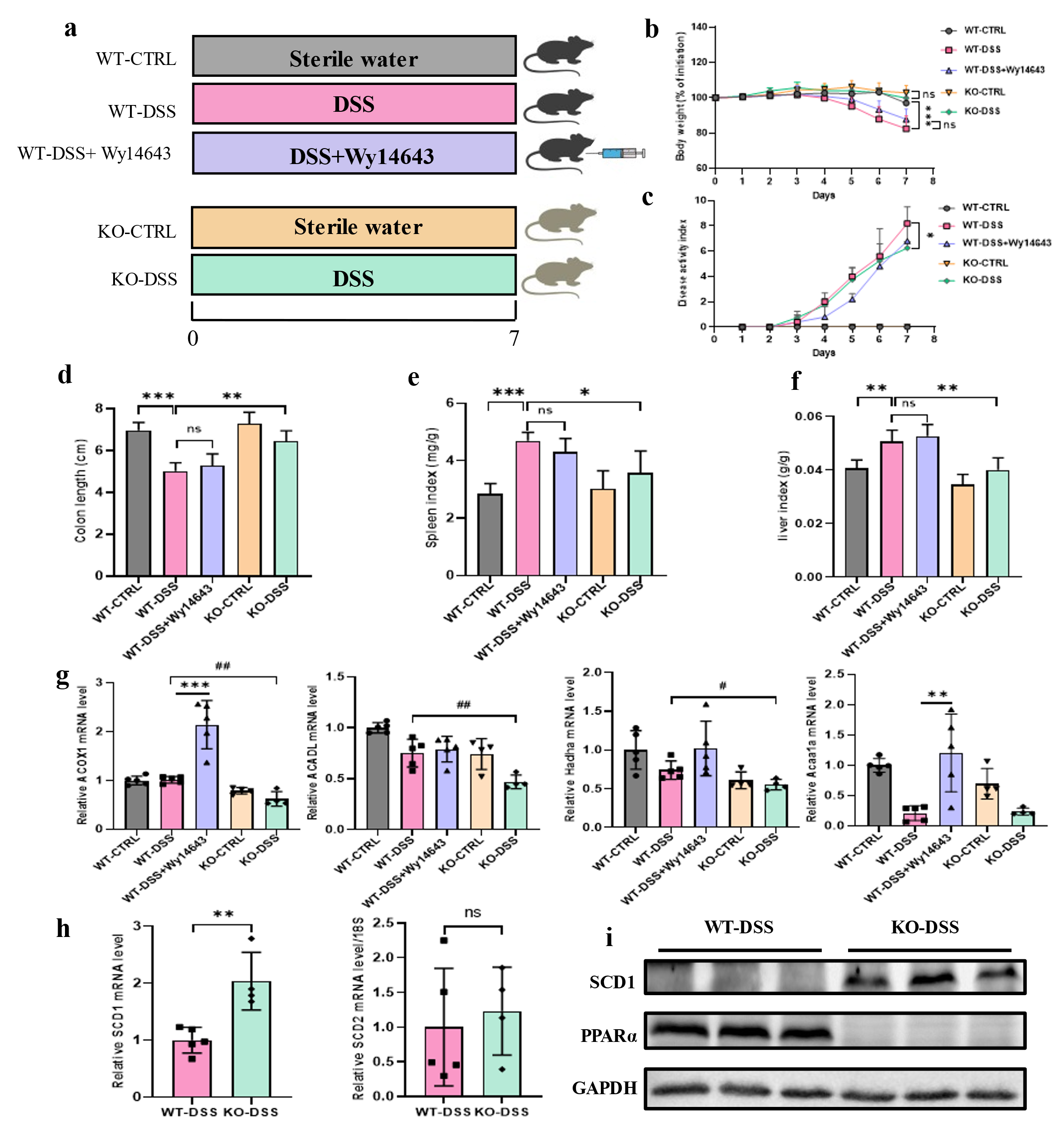


**Figure S7 PPARα KO-induced upregulation of hepatic SCD1 expression exerts potential benefits for DSS-induced colitis. a** Schematic diagram of mouse experiment. **b, c** Body weight changes and Disease activity index (DAI) across the experiment (WT: n=5, KO: n=4). **d-f** Colon length, spleen and liver (Ratio of spleen/liver weight to body weight) indices (WT: n=5, KO: n=4). **g** Relative expression of ACOX1, ACDAL, Hadha and Acaa1 in mice liver (WT: n=5, KO: n=4). **h** Relative expression of hepatic SCD1 and SCD2 (WT-DSS: n=5, KO-DSS: n=4). **i** Protein expression of hepatic SCD1 (n=3). *, #*p* < 0.05, **, ##*p* < 0.01, ***, ###*p* < 0.001.

**Table S1. Summary of typical altered metabolites by PSM in Serum**

| **Metabolites** | **Mass (m/z)**  **[M+H]^+^ / [M-H]^-^** | **RT** | **Main fragments** | **Mode** |
| --- | --- | --- | --- | --- |
| LysoPC 18:1 | 522.3529 | 10.95 | 339;184;104; | ESI+ |
| LysoPC 18:2 | 520.3377 | 10.22 | 337;184;86 | ESI+ |
| LysoPC 18:3 | 518.3223 | 9.74 | 184 | ESI+ |
| LysoPC 20:3 | 546.3551 | 10.65 | 524;184;86 | ESI+ |
| LysoPC 20:4 | 544.3380 | 10.24 | 184;104;86 | ESI+ |
| LysoPE 18:1 | 480.3070 | 10.89 | 339 | ESI+ |
| LysoPE 18:2 | 478.2914 | 10.16 | 337 | ESI+ |
| Indole-3-propionic acid* | 190.0858 | 6.713 | 170;132;55 | ESI+ |
| N1-Acetylspermidine | 188.1753 | 0.781 | 171;100;72 | ESI+ |
| Methylhistidine | 170.0922 | 0.789 | 152;126;124;109 | ESI+ |
| Carnitine* | 162.1119 | 0.828 | 103;85;60 | ESI+ |
| C2 Carnitine* | 204.1229 | 1.02 | 145;85;60 | ESI+ |
| C18:2 Carnitine | 424.3415 | 10.25 | 365;85;69;60 | ESI+ |
| C18:1 Carnitine | 426.3569 | 10.77 | 85 | ESI+ |
| C7-DC Carnitine* | 304.1740 | 3.581 | 285;245;85 | ESI+ |
| D/L-Lactic acid* | 89.0248 | 1.025 | 71;59 | ESI- |
| Taurodeoxycholic acid* | 498.2902 | 7.846 | 124;106;79 | ESI- |
| Deoxycholic acid* | 391.2852 | 10.07 | 355;345;327;94 | ESI- |

*Metabolites identified by authentic standards

**Table S2. Summary of typical altered metabolites by PSM in liver**

| **Metabolites** | **Mass (m/z)**  **[M+H]^+^/ [M-H]^-^** | **RT** | **Main fragments** | **Mode** |
| --- | --- | --- | --- | --- |
| LPC 16:1 | 494.3233 | 9.84 | 476;184;104;86 | ESI+ |
| LPC 18:1 | 522.3545 | 10.96 | 339;184;104 | ESI+ |
| LPC 18:2 | 520.3394 | 10.04 | 337;184;86 | ESI+ |
| LPC 20:3 | 546.3537 | 10.48 | 528;184;86 | ESI+ |
| LPC 20:4 | 544.3399 | 10.08 | 184;104;86 | ESI+ |
| LPC P-18:0 | 508.3749 | 11.30 | 490;467;184;104 | ESI+ |
| LPE 16:1 | 450.2622 | 9.75 | 253;214;196;140 | ESI- |
| LPE 18:0 | 480.3085 | 11.76 | 283;196;140;78 | ESI- |
| LPE 18:1 | 480.3067 | 10.87 | 339 | ESI+ |
| LPE 18:2 | 478.2916 | 9.96 | 337 | ESI+ |
| LPE 20:4 | 502.2934 | 10.01 | 330;269;203;91 | ESI+ |
| LPE 22:6 | 526.2914 | 9.99 | 354;341;216;97 | ESI+ |
| D/L-Valine* | 170.0922 | 0.78 | 152;126;124;109 | ESI+ |
| N-Acetylleucine | 172.0979 | 5.21 | 130;128;58 | ESI- |
| N-Isobutyrylglycine | 144.0668 | 2.91 | 100;74 | ESI- |
| Adipic acid | 145.0508 | 3.43 | 101;83 | ESI- |
| Citramalic acid | 147.0300 | 1.02 | 129;103;85;57 | ESI- |
| Citraconic acid | 129.0196 | 1.02 | 85 | ESI- |
| Gluconic acid | 195.0514 | 0.81 | 129;99;75;59 | ESI- |
| D/L-Lactic acid* | 89.0245 | 1.02 | 71;59 | ESI- |
| Glutathione (reduced)* | 308.0902 | 1.02 | 233;179;162;76 | ESI+ |
| Taurodeoxycholic acid* | 500.3030 | 7.92 | 464;339;126 | ESI+ |
| Xanthosine | 283.0686 | 2.59 | 151 | ESI- |
| Carnitine* | 162.1123 | 0.81 | 103;85;60 | ESI+ |
| C2 Carnitine* | 204.1232 | 1.02 | 145;85;60 | ESI+ |
| C3 Carnitine | 218.1382 | 2.12 | 159;85;60 | ESI+ |
| C5-OH Carnitine | 262.1646 | 2.46 | 203;85;60 | ESI+ |
| C6-DC Carnitine | 290.1595 | 2.99 | 213;85;60 | ESI+ |
| C7-DC Carnitine* | 304.1751 | 3.53 | 285;245;85 | ESI+ |
| C14 Carnitine | 372.3098 | 9.64 | 313;85 | ESI+ |
| C14-OH Carnitine | 388.3043 | 8.60 | 329;85 | ESI+ |
| C16 Carnitine***** | 400.3404 | 10.52 | 341;85 | ESI+ |
| C16-OH Carnitine | 416.3363 | 9.56 | 357;339;85 | ESI+ |
| C16:1 Carnitine | 398.3251 | 10.32 | 339;85 | ESI+ |
| C16:2 Carnitine | 396.3103 | 9.43 | 337;85;60 | ESI+ |
| C16:2-OH Carnitine | 412.3054 | 8.54 | 85 | ESI+ |
| C18-OH Carnitine | 444.3669 | 10.45 | 365;316;85 | ESI+ |
| C18:2 Carnitine | 424.3414 | 10.20 | 365;85;69;60 | ESI+ |
| C20:4 Carnitine | 448.3422 | 10.20 | 389;85 | ESI+ |
| C22:6 Carnitine | 472.3406 | 10.15 | 413;85 | ESI+ |
| Stearic acid | 283.2640 | 11.32 | 73 | ESI- |
| D/L-Lysine* | 147.1125 | 0.70 | 130;84 | ESI- |
| D/L-Proline* | 116.0706 | 0.69 | 70 | ESI- |
| Citrulline | 176.1025 | 0.81 | 159;113;70 | ESI+ |
| Ornithine | 133.0971 | 0.69 | 116;70 | ESI+ |
| Adipoylglycine | 202.0719 | 2.49 | 184;160;140;116;96 | ESI- |
| Pyroglutamic acid | 128.0354 | 1.02 | 82 | ESI- |
| N-Methylglutamic acid | 160.0615 | 0.88 | 142;116;98 | ESI- |
| N-Acetylglutamic acid | 188.0564 | 1.02 | 144;128;102;59 | ESI- |
| D/L-Glutamic acid* | 146.0459 | 1.02 | 128;102 | ESI- |
| Methylsuccinic acid | 131.0351 | 2.54 | 113;87;69 | ESI- |
| 3-Methyladipic acid | 159.0665 | 3.53 | 141;115;97 | ESI- |
| Pantothenic acid | 218.1036 | 3.10 | 146;88;71 | ESI- |
| N-Acetylneuraminic acid | 160.0615 | 0.88 | 142;116;98 | ESI- |
| Gly-Leu | 187.1091 | 3.21 | 130;73 | ESI- |
| O-Phosphocolamine | 140.0120 | 0.77 | 78 | ESI- |
| Acetylcholine* | 146.1173 | 0.87 | 87;60 | ESI+ |
| sphinganine | 302.3043 | 9.75 | 284;266;254;240;95;81 | ESI+ |
| sphingosine | 300.2892 | 9.51 | 282;265;264;252;211 | ESI+ |
| Corticosterone | 347.2202 | 7.52 | 329;311;269 | ESI+ |
| Dihydrocoticosterone | 349.2400 | 6.74 | 311;313;271 | ESI+ |
| 11-dehydrocorticosterone | 345.2100 | 7.28 | 327;309 | ESI+ |

*****Metabolites identified by authentic standards

**Table S3. Summary of typical altered lipid metabolites by PSM in liver**

| **Metabolites** | **Mass (m/z)**  **[M+H]^+^ / [M+H-H_2_O]^+^** | **RT** | **Transition** | **Mode** |
| --- | --- | --- | --- | --- |
| LPC 14:0 | 468.3086 | 0.97 | 468.30 → 184.07 | ESI+ |
| LPC 15:0 | 482.3240 | 1.12 | 462.32 → 184.07 | ESI+ |
| LPC 16:0 | 496.3377 | 1.31 | 496.33 → 184.07 | ESI+ |
| LPC 17:0 | 510.3548 | 1.55 | 510.35 → 184.07 | ESI+ |
| LPC 18:0 | 524.3712 | 1.88 | 524.37 → 184.07 | ESI+ |
| LPC 19:0 | 538.3883 | 2.30 | 538.33 → 184.07 | ESI+ |
| LPC 20:0 | 552.4032 | 2.82 | 552.40 → 184.07 | ESI+ |
| LPC 22:0 | 580.4320 | 3.95 | 580.43 → 184.07 | ESI+ |
| LPC 23:0 | 594.4496 | 4.52 | 594.49 → 184.07 | ESI+ |
| LPC 24:0 | 608.4663 | 5.20 | 608.46 → 184.07 | ESI+ |
| LPC 16:1 | 494.3323 | 1.02 | 494.33 → 184.07 | ESI+ |
| LPC 17:1 | 508.3404 | 1.18 | 508.34 → 184.07 | ESI+ |
| LPC 18:1 | 522.3559 | 1.36 | 522.35 → 184.07 | ESI+ |
| LPC 18:2 | 520.3376 | 1.04 | 520.33 → 184.07 | ESI+ |
| LPC 18:3 | 518.3228 | 0.92 | 518.32 → 184.07 | ESI+ |
| LPC 19:1 | 536.3737 | 1.62 | 536.37 → 184.07 | ESI+ |
| LPC 20:1 | 550.3859 | 1.95 | 550.38 → 184.07 | ESI+ |
| LPC 20:2 | 548.3707 | 1.46 | 548.37 → 184.07 | ESI+ |
| LPC 20:3 | 546.3552 | 1.14 | 546.35 → 184.07 | ESI+ |
| LPC 22:1 | 578.4178 | 2.86 | 578.41 → 184.07 | ESI+ |
| LPC 24:1 | 606.4470 | 3.96 | 606.44 → 184.07 | ESI+ |
| LPC 24:2 | 604.4330 | 3.07 | 604.43 → 184.07 | ESI+ |
| LPC O-16:0 | 482.3611 | 1.54 | 482.36 → 184.07 | ESI+ |
| LPC O-18:0 | 510.3930 | 2.27 | 510.39 → 184.07 | ESI+ |
| LPC O-20:0 | 538.4238 | 3.40 | 538.42 → 184.07 | ESI+ |
| LPC O-22:0 | 566.4551 | 4.51 | 566.45 → 184.07 | ESI+ |
| LPC O-24:0 | 594.4840 | 6.00 | 594.48 → 184.07 | ESI+ |
| LPC O-16:1 | 480.3509 | 1.47 | 480.35 → 184.07 | ESI+ |
| LPC O-18:1 | 508.3758 | 1.61 | 508.37 → 184.07 | ESI+ |
| LPC O-18:2 | 506.3605 | 1.24 | 506.36 → 184.07 | ESI+ |
| LPC O-19:3 | 518.3670 | 0.95 | 518.36 → 184.07 | ESI+ |
| LPC O-21:2 | 548.4071 | 1.41 | 548.40 → 184.07 | ESI+ |
| LPC O-24:1 | 592.4716 | 4.49 | 592.47 → 184.07 | ESI+ |
| LPE 16:0 | 454.2934 | 1.38 | 454.29 → 313.27 | ESI+ |
| LPE 17:0 | 468.3083 | 1.66 | 468.30 → 327.28 | ESI+ |
| LPE 18:0 | 482.3240 | 2.01 | 482.32 → 341.30 | ESI+ |
| LPE 18:1 | 480.3083 | 1.46 | 480.30 → 339.28 | ESI+ |
| LPE 18:2 | 478.2903 | 1.11 | 478.29 → 337.27 | ESI+ |
| LPE 20:4 | 502.2922 | 1.06 | 502.29 → 361.27 | ESI+ |
| LPE 22:6 | 526.2926 | 1.00 | 526.29 → 385.27 | ESI+ |
| LPE O-16:1 | 438.2972 | 1.59 | 438.29 → 266.28 | ESI+ |
| LPE O 18:1 | 466.3302 | 2.35 | 466.33 → 294.31 | ESI+ |
| LPE O-18:2 | 464.3120 | 2.01 | 464.31 → 310.31 | ESI+ |
| LPE O-20:1 | 494.3613 | 3.49 | 494.36 → 322.34 | ESI+ |
| Cer (18:1;2O/16:0) | 520.5070 | 10.43 | 520.50 → 264.26 | ESI+ |
| Cer (18:1;2O/18:0) | 548.5413 | 13.61 | 548.54 → 264.26 | ESI+ |
| Cer (18:1;2O/20:0) | 576.5712 | 14.44 | 576.57 → 264.27 | ESI+ |
| Cer (18:1;2O/22:0) | 622.6113 | 15.07 | 622.61 → 264.26 | ESI+ |
| Cer (18:1;2O/22:1) | 620.5970 | 14.43 | 620.59 → 264.27 | ESI+ |
| Cer (18:1;2O/24:0) | 632.6351 | 15.63 | 632.63 → 264.26 | ESI+ |
| Cer (18:1;2O/24:1) | 630.6181 | 15.03 | 630.61 → 264.26 | ESI+ |
| HexCer (18:1;2O/16:0) | 700.5702 | 8.25 | 700.57 → 264.27 | ESI+ |
| HexCer (18:1;2O/18:0) | 728.6035 | 11.14 | 728.60 → 264.27 | ESI+ |
| HexCer (18:1;2O/20:0) | 756.6354 | 13.79 | 756.63 → 264.27 | ESI+ |
| HexCer (18:1;2O/22:0) | 784.6660 | 14.52 | 784.66 → 264.27 | ESI+ |
| HexCer (18:1;2O/22:1) | 782.6480 | 13.78 | 782.64 → 264.26 | ESI+ |
| HexCer (18:1;2O/24:0) | 812.6931 | 15.11 | 812.69 → 264.26 | ESI+ |
| HexCer (18:1;2O/24:1) | 810.6802 | 14.48 | 810.68 → 264.26 | ESI+ |
| SM (18:1;2O/16:0) | 703.5727 | 7.32 | 703.57 → 184.07 | ESI+ |
| SM (18:1;2O/18:0) | 731.6055 | 9.89 | 731.60 → 184.07 | ESI+ |
| SM (18:1;2O/20:0) | 759.6355 | 13.26 | 759.63 → 184.07 | ESI+ |
| SM (18:2;2O/22:0) | 785.6513 | 13.54 | 785.65 → 184.07 | ESI+ |
| SM (18:1;2O/22:1) | 785.6517 | 13.20 | 785.65 → 184.07 | ESI+ |
| SM (18:1;2O/24:0) | 815.7001 | 14.90 | 815.70 → 184.07 | ESI+ |
| SM (18:1;2O/24:1) | 813.6817 | 14.23 | 813.68 → 184.07 | ESI+ |

**Table S4. Sequences of real time PCR primers**

| **Primers** | **Sequence (5'-3')** | **Species** |
| --- | --- | --- |
| IL6 | Forward primer: CGGAGAGGAGACTTCACAGAGGA  Reverse primer: TTTCCACGATTTCCCAGAGAACA | Mouse |
| IL-1β | Forward primer: CCCTGCAGCTGGAGAGTGTGGA  Reverse primer: TGTGCTCTGCTTGTGAGGTGCTG | Mouse |
| IL-17A | Forward primer: TCAGCGTGTCCAAACACTGAG  Reverse primer: CGCCAAGGGAGTTAAAGACTT | Mouse |
| TNFα | Forward primer: CCACCACGCTCTTCTGTCTAC  Reverse primer: AGGGTCTGGGCCATAGAACT | Mouse |
| iNOS | Forward primer: GTTCTCAGCCCAACAATACAAGA  Reverse primer: GTGGACGGGTCGATGTCAC | Mouse |
| Cxcl2 | Forward primer: CCAACCACCAGGCTACAGG  Reverse primer: GCGTCACACTCAAGCTCTG | Mouse |
| CCL2 | Forward primer: AGGTCCCTGTCATGCTTCTG  Reverse primer: GGGATCATCTTGCTGGTGAA | Mouse |
| Gclc | Forward primer: GGCGATGTTCTTGAGACTCTGC  Reverse primer: TTCCTTCGATCATGTAACTCCCATA | Mouse |
| GPX2 | Forward primer: GCCTCAAGTATGTCCGACCTG  Reverse primer: GGAGAACGGGTCATCATAAGGG | Mouse |
| SOD1 | Forward primer: AAGCGGTGAACCAGTTGTGTT  Reverse primer: AGCCTTGTGTATTGTCCCCATACT | Mouse |
| TGFβ | Forward primer: CTCCCGTGGCTTCTAGTGC  Reverse primer: GCCTTAGTTTGGACAGGATCTG | Mouse |
| MMP9 | Forward primer: GCAGAGGCATACTTGTACCG  Reverse primer: TGATGTTATGATGGTCCCACTTG | Mouse |
| PAI-1 | Forward primer: TCTGGGAAAGGGTTCACTTTACC  Reverse primer: GACACGCCATAGGGAGAGAAG | Mouse |
| BSEP | Forward primer: CCAGAACATGACAAACGGAA  Reverse primer: AAGGACAGCCACACCAACTC | Mouse |
| NTCP | Forward primer: AGGGGGACATGAACCTCAG  Reverse primer: TCCGTCGTAGATTCCTTTGC | Mouse |
| MPR2 | Forward primer: GTGTGGATTCCCTTGGGCTTT  Reverse primer: CACAACGAACACCTGCTTGG | Mouse |
| OATP1 | Forward primer: ACTCCCATAATGCCCTTGG  Reverse primer: TAATCGGGCCAACAATCTTC | Mouse |
| CerS2 | Forward primer: AAGTGGGAAACGGAGTAGCG  Reverse primer: ACAGGCAGCCATAGTCGTTC | Mouse |
| CerS6 | Forward primer: AAGCCAATGGACCACAAACT  Reverse primer: TGCTTGGAGAGCCCTTCTAAT | Mouse |
| SMPD1 | Forward primer: GTTACCAGCTGATGCCCTTC  Reverse primer: AGGCTACTGTCTTGGCTAGGA | Mouse |
| SMPD2 | Forward primer: CTCCAGCCATGAAGCTCAAC  Reverse primer: TTCAGAAAGTCTCCCAAGCG | Mouse |
| SMPD3 | Forward primer: CCTGACCAGTGCCATTCTTT  Reverse primer: AGAAACCCGGTCCTCGTACT | Mouse |
| SMS | Forward primer: CACAGCACGCTCGACTTCAA  Reverse primer: TGCCATTCTTGTTCGTGTAAGTT | Mouse |
| Sphk1 | Forward primer: AAAATACTGAGAAACTCGGTCGG  Reverse primer: GCATCGCTTCTTAAAGTCCAGA | Mouse |
| Sphk2 | Forward primer: CACGGCGAGTTTGGTTCCTA  Reverse primer: CTTCTGGCTTTGGGCGTAGT | Mouse |
| SGPL1 | Forward primer: CTGAAGGACTTCGAGCCTTATTT  Reverse primer: ACTCCACGCAATGAGCTGC | Mouse |
| Cerk | Forward primer: AAATCCGTTCGCATTCACAGT  Reverse primer: CAAGTGCTTCGGTCTTGAAGT | Mouse |
| ZO-1 | Forward primer: ACCCGAAACTGATGCTGTGGATAG  Reverse primer: AAATGGCCGGGCAGAACTTGTGTA | Mouse |
| Occludin | Forward primer: GGAGGACTGGGTCAGGGAATA  Reverse primer: CGTCGTCTAGTTCTGCCTGT | Mouse |
| Claudin | Forward primer: GGCTGTTAGGCACATCCAT  Reverse primer: TGGCACCAACATAGGAACTC | Mouse |
| PV-1 | Forward primer: GCTGGTACTACCTGCGCTATT  Reverse primer: CCTGTGAGGCAGATAGTCCA | Mouse |
| LXRα | Forward primer: CTCAATGCCTGATGTTTCTCCT  Reverse primer: TCCAACCCTATCCCTAAAGCAA | Mouse |
| SREBF1 | Forward primer: GGAGCCATGGATTGCACATT  Reverse primer: GCTTCCAGAGAGGAGGCCAG | Mouse |
| Acaca | Forward primer: ATGGGCGGAATGGTCTCTTTC  Reverse primer: TGGGGACCTTGTCTTCATCAT | Mouse |
| FASN | Forward primer: AAGTTGCCCGAGTCAGAGAACC  Reverse primer: ATCCATAGAGCCCAGCCTTCCATC | Mouse |
| SCD1 | Forward primer: TTCTTGCGATACACTCTGGTGC  Reverse primer: CGGGATTGAATGTTCTTGTCGT | Mouse |
| SCD2 | Forward primer: GCATTTGGGAGCCTTGTACG  Reverse primer: AGCCGTGCCTTGTATGTTCTG | Mouse |
| FADS2 | Forward primer: GATGGCTGCAACATGACTATGG  Reverse primer: GCTGAGGCACCCTTTAAGTGG | Mouse |
| ELOVL5 | Forward primer: ATGGAACATTTCGATGCGTCA  Reverse primer: GTCCCAGCCATACAATGAGTAAG | Mouse |
| DGAT1 | Forward primer: GTGCCATCGTCTGCAAGATTC  Reverse primer: GCATCACCACACACCAATTCAG | Mouse |
| DGAT2 | Forward primer: CCGAACACTCTCCATCTTACAGT  Reverse primer: ACAAGGGCCATAAGGTAGTGA | Mouse |
| Lpcat3 | Forward primer: AGCCTTAACAAGTTGGCGAC  Reverse primer: AGCCTTAACAAGTTGGCGAC | Mouse |
| MOGAT1 | Forward primer: TGGTGCCAGTTTGGTTCCAG  Reverse primer: TGCTCTGAGGTCGGGTTCA | Mouse |
| ACSL4 | Forward primer: CTCACCATTATATTGCTGCCTGT  Reverse primer: TCTCTTTGCCATAGCGTTTTTCT | Mouse |
| CD36 | Forward primer: TCCTCTGACATTTGCAGGTCTATC  Reverse primer: AAAGGCATTGGCTGGAAGAA | Mouse |
| Fabp1 | Forward primer: TTTCAAAGGCATAAAGTCCGTG  Reverse primer: CTTGCTGACTCTCTTGTAGACA | Mouse |
| Fabp4 | Forward primer: AAGGTGAAGAGCATCATAACCCT  Reverse primer: TCACGCCTTTCATAACACATTCC | Mouse |
| PPARα | Forward primer: CCCAAGGGAGGAATAGCTTCT  Reverse primer: CTCTGCGATGCGGTTCCAA | Mouse |
| Acox1 | Forward primer: TGGTATGGTGTCGTACTTGAATGAC  Reverse primer: AATTTCTACCAATCTGGCTGCAC | Mouse |
| MCAD | Forward primer: GCGAGCAGAAATGAAACTCC  Reverse primer: AGCTCTAGACGAAGCCACGA | Mouse |
| Acaa1a | Forward primer: TCTCCAGGACGTGAGGCTAAA  Reverse primer: CGCTCAGAAATTGGGCGATG | Mouse |
| Acot1 | Forward primer: ATGGCAGCAGCTCCAGACTT  Reverse primer: CCCAACCTCCAAACCATCAT | Mouse |
| HADHA | Forward primer: AAGGGGATGTGGCAGTTATT  Reverse primer: ACTCCTGATTTGGTCGTTGG | Mouse |
| ACADL | Forward primer: TCTTTTCCTCGGAGCATGACA  Reverse primer: GACCTCTCTACTCACTTCTCCAG | Mouse |
| Hmgcs2 | Forward primer: GGCGGGTCCTGCAAGTGAAGA  Reverse primer: GGGGAGCAGGAGGGATTGTAGAAA | Mouse |
| Cpt1 | Forward primer: CTCCGCCTGAGCCATGAAG  Reverse primer: CACCAGTGATGATGCCATTCT | Mouse |
| Cpt2 | Forward primer: CAGCACAGCATCGTACCCA  Reverse primer: TCCCAATGCCGTTCTCAAAAT | Mouse |
| ACSL1 | Forward primer: CCTCTAATGGCTGCAAGGCTA  Reverse primer: CCAGGTGATAGAAGTCCCATCT | Mouse |
| ABCG1 | Forward primer: CTTTCCTACTCTGTACCCGAGG  Reverse primer: CGGGGCATTCCATTGATAAGG | Mouse |
| CYP7A1 | Forward primer: GGGAATGCCATTTACTTGGA  Reverse primer: GTCCGGATATTCAAGGATGC | Mouse |
| CYP27A1 | Forward primer: GGAAGGTGCCCCAGAACAA  Reverse primer: GCGCAGGGTCTCCTTAATCA | Mouse |
| LPL | Forward primer: GGGAGTTTGGCTCCAGAGTTT  Reverse primer: TGTGTCTTCAGGGGTCCTTAG | Mouse |
| *L.johnsonii* | Forward primer: TCGAGCGAGCTTGCCTAGATGA  Reverse primer: TCCGGACAACGCTTGCCACC | Bacteria |
| *L.murinus* | Forward primer: TCGAACGAAACTTCTTTATCACC  Reverse primer: CGTTCGCCACTCAACTCTTT | Bacteria |
| *L.reuteri* | Forward primer: YCACCGCTACACATGRAGTTCCACT  Reverse primer: GATCCATCGTCAATCAGG | Bacteria |
| *Eubacteria* | Forward primer: CGGCAACGAGCGCAACCC  Reverse primer: CCATTGTAGCACGTGTGTAG | Bacteria |
| GAPDH | Forward primer: TTGATGGCAACAATCTCCAC  Reverse primer: CGTCCCGTAGACAAAATGGT | Mouse |
| β-actin | Forward primer: TGTTACCAACTGGGACGACA  Reverse primer: CTGGGTCATCTTTTCACGGT | Mouse |
| HRPT | Forward primer: TCAGTCAACGGGGGACATAAA  Reverse primer: GGGGCTGTACTGCTTAACCAG | Mouse |
| TLR2 | Forward primer: CTCTTCAGCAAACGCTGTTCT  Reverse primer: GGCGTCTCCCTCTATTGTATTG | Mouse |
| TLR4 | Forward primer: TCCATTTCAGCTCTGCCTTCAC  Reverse primer: ACACCACAACAATCACCTTTCG | Mouse |
| SREBF1 | Forward primer: GGAGGGGTAGGGCCAACGGCCT  Reverse primer: CATGTCTTCGAAAGTGCAATCC | Human |
| FASN | Forward primer: AAGGACCTGTCTAGGTTTGATGC  Reverse primer: TGGCTTCATAGGTGACTTCCA | Human |
| SCD1 | Forward primer: GCCCCTCTACTTGGAAGACGA  Reverse primer: AAGTGATCCCATACAGGGCTC | Human |
| GAPDH | Forward primer: GGAGCGAGATCCCTCCAAAAT  Reverse primer: GGCTGTTGTCATACTTCTCATGG | Human |
